# Digital literacy in undergraduate pharmacy education: a scoping review

**DOI:** 10.1093/jamia/ocad223

**Published:** 2023-12-05

**Authors:** Mashael Alowais, Georgina Rudd, Victoria Besa, Hamde Nazar, Tejal Shah, Clare Tolley

**Affiliations:** School of Pharmacy, Newcastle University, Newcastle upon Tyne, NE1 7RU, United Kingdom; Department of Pharmacy Practice, Unaizah College of Pharmacy, Qassim University, Unaizah, 51911, Saudi Arabia; School of Pharmacy, Newcastle University, Newcastle upon Tyne, NE1 7RU, United Kingdom; School of Pharmacy, Newcastle University, Newcastle upon Tyne, NE1 7RU, United Kingdom; School of Pharmacy, Newcastle University, Newcastle upon Tyne, NE1 7RU, United Kingdom; School of Computing, Newcastle University, Newcastle upon Tyne, NE4 5TG, United Kingdom; School of Pharmacy, Newcastle University, Newcastle upon Tyne, NE1 7RU, United Kingdom

**Keywords:** digital health, digital literacy, education, pharmacy students, electronic health records

## Abstract

**Objectives:**

Conduct a scoping review to identify the approaches used to integrate digital literacy into undergraduate pharmacy programs across different countries, focusing on methods for education, training, and assessment.

**Materials and methods:**

Following the Joanna Briggs Institute methodology, we searched 5 electronic databases in June 2022: MEDLINE (Ovid), PubMed, Embase, Scopus, and CINAHL. Three independent reviewers screened all articles; data extraction was conducted by 2 reviewers. Any discrepancies were arbitrated by 2 additional reviewers.

**Results:**

Out of 624 articles, 57 were included in this review. Educational and training approaches for digital literacy in undergraduate pharmacy programs encompassed a theoretical understanding of health informatics, familiarization with diverse digital technologies, and applied informatics in 2 domains: patient-centric care through digital technologies, and the utilization of digital technologies in interprofessional collaboration. Blended pedagogical strategies were commonly employed. Assessment approaches included patient plan development requiring digital information retrieval, critical appraisal of digital tools, live evaluations of telehealth skills, and quizzes and exams on health informatics concepts. External engagement with system developers, suppliers, and other institutes supported successful digital literacy education.

**Discussion and conclusion:**

This scoping review identifies various learning objectives, teaching, and assessment strategies to incorporate digital literacy in undergraduate pharmacy curricula. Recommendations include acknowledging the evolving digital health landscape, ensuring constructive alignment between learning objectives, teaching approach and assessments, co-development of digital literacy courses with stakeholders, and using standardized guidelines for reporting educational interventions. This study provides practical suggestions for enhancing digital literacy education in undergraduate pharmacy programs.

## Introduction

Digital health is an umbrella term that refers to “the use of technologies for healthcare”.^1^ It encompasses a wide range of fields, including electronic health (eHealth), wearable and mobile health technology (mHealth), and emerging areas, such as big data, genomics, and artificial intelligence (AI).^1–3^ Digital health technologies (DHTs) have been increasingly important and beneficial in healthcare, with the coronavirus disease 2019 pandemic further accelerating their roles particularly in areas of telemedicine and remote care.^4–6^

DHTs have been increasingly adopted in pharmacy practice, including electronic patient records (EPRs), clinical decision support (CDS) systems, e-prescribing, and robotic medicine dispensing, to support delivery of patient care.^7^^,^^8^ Pharmacists are using DHT products and services to optimize patient care, for example, to document interventions, perform assessments, patient education, and monitor patients.^9^ Although the shift toward digital health promises substantial benefits, achieving these will require a digitally literate healthcare workforce.^5^^,^^10^ Furthermore, due to the rapid advancement in the use of technology around the world, the pharmacy profession cannot wait for the gradual incorporation of digital technologies into practice and education.^7^

In the United States, the Digital Nation initiative was launched in 2010, to promote digital literacy and inclusion, with the Accreditation Council for Pharmacy Education (ACPE) developing standards for Digital Health in 2016.^11^ These standards were designed to ensure that future pharmacists were equipped to utilize DHTs effectively while providing patient care.^12^ In the United Kingdom, NHS England supports workforce transformation and defines digital literacy as “those capabilities that fit someone for living, learning, working, participating and thriving in a digital society”.^13^ In 2021, Health Education England (HEE) (*now part of NHS England*) published a guidance document on digital literacy for the pharmacy workforce. The report focused on major aspects of digital transformation, including how to improve the pharmacy workforce’s digital literacy and align to the National Health Service’s (NHS) Long-Term Plan and the NHS People’s Plan.^13^ Despite these efforts, when the International Pharmaceutical Federation reviewed digital health in pharmacy education, they found that a large proportion of pharmacy schools do not incorporate digital health education in their curricula.^14^

Evidence suggests that improving the digital health literacy of the pharmacy workforce should be comprehensive, starting with undergraduate education and continuing through postgraduate training and beyond.^5^ As digital literacy is still a relatively new concept in pharmacy education, there is a need to systematically explore the current evidence to identify successful approaches and recommendations for integrating digital literacy into education. A preliminary search of MEDLINE, the Cochrane Database of Systematic Reviews, and Joanna Briggs Institute (JBI) Evidence Synthesis was conducted, and no current systematic reviews on the topic were identified.

Through a scoping review, we aim to address the research question: “What strategies for education, training, and assessing digital literacy are used for undergraduate pharmacy students and trainees globally?” The objectives of the review are as follows: (1) explore educational and training approaches for equipping pharmacy students and trainees with digital literacy skills, (2) identify digital competencies/capabilities included in the curricula, (3) identify the learning outcomes integrated into the curricula, (4) investigate pedagogical methods used to deliver digital literacy content, (5) explore methods for assessing digital literacy in pharmacy students and trainees, and (6) identify factors that contribute to or hinder the effectiveness of implementing digital literacy in pharmacy programs. We further hope to identify areas for improvement and future research.

## Methods

The scoping review was conducted in accordance with the JBI methodology for scoping reviews.^15^ A protocol detailing the proposed method was registered in Open Science Framework: 10.17605/OSF.IO/T6BP5, which is summarized in the following sections.

### Eligibility criteria

Table 1 outlines the eligibility criteria for the review which are framed around participants, concept, context (PCC) and types of sources.

**Table 1.** Eligibility criteria for the scoping review.

PCC	Description
Participants	Studies are selected if they include undergraduate pharmacy students or foundation trainee/preregistration pharmacists. Qualified pharmacists will be excluded*.*
Concept	Studies will be considered if they include digital literacy in respect to Curriculum development, a description or list of competencies or learning outcomes, or recommendations on educational approaches. A description of the training program or training approaches. Digital literacy assessment methods. Digital literacy curriculum evaluations or examining the impact or outcomes of digital literacy implementation.
Context	Worldwide studies.
Types of sources	All study types and methods were considered. Given that the concept of health informatics (HI) was broadly adopted from 1995, papers prior to this date will be excluded.^16^

### Search strategy

**Figure 1.**
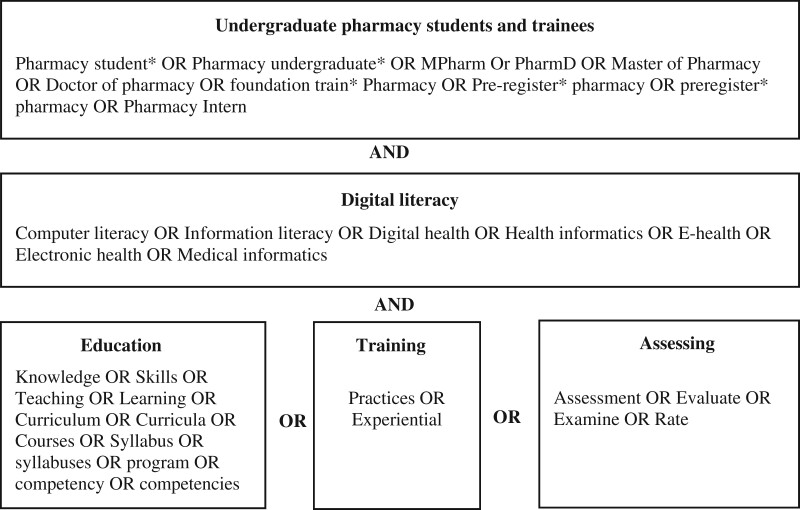
Elements of the scoping review search strategy.

The search strategy commenced in June 2022, an initial limited search of MEDLINE (Ovid) to identify keywords contained in the titles and abstracts or index terms of relevant articles. A comprehensive search was then conducted in EMBASE, PubMed, Scopus, CINAHL, MEDLINE (Ovid), and key journals in the research area, for example, *American Journal of Pharmaceutical Education*, *BMC Medical Education*, *Medical Education*, and *Medical Teacher* with keywords and index terms on concepts of digital literacy, education, training, assessment, and undergraduate pharmacy students and trainees, that were slightly modified to fit each database’s requirements (Figure 1). No search limitations (eg, dates) were applied to the search. The detailed search strategy used for MEDLINE (Ovid), which was the basis for all other searches is included in Table S1. Details of the search strategies used for the other databases are presented in Table S2. Finally, the reference lists of all included sources were screened to identify additional studies. Studies published in English, prior to 1995 were excluded.

### Study selection

All identified studies were collated and uploaded into EndNote 20.2 (Clarivate Analytics, PA, USA), where duplicates were removed using the “remove duplicates” function. A secondary manual check was also conducted to ensure complete removal of all duplicates. The study selection was performed in 2 phases and was conducted by 3 reviewers (M.A., G.R., V.B.). Initially, titles and abstracts of the identified studies were screened independently by the reviewers, based on the predefined inclusion and exclusion criteria. Included studies then progressed to the full-text review phase and were further independently screened by all reviewers. The screening process was facilitated using Microsoft Excel, reviewers documented their decisions noting any reasons for exclusion where relevant. Throughout the evidence selection process, periodic meetings were held to discuss divergent opinions and reach collective decisions. Any disagreements that arose were resolved through discussion, or with additional reviewers (C.T., H.N.).

### Data extraction

A pilot was conducted using a select sample of approximately 10 studies to test the preliminary data extraction form. Based on the insights from this pilot, modifications were made to the form, now provided in Table S3. The data extraction table included detailed criteria such as PCC, study methods, and key findings in line with the review’s aim. Two independent reviewers (M.A. and G.R.) conducted the data extraction on half the included studies. The lead author further reviewed and double-checked data extraction across all included studies. Where discrepancies in extraction arose, they were discussed between both reviewers (M.A. and G.R.), with further input from C.T. where necessary to reach consensus.

### Data analysis

A thematic analysis was conducted to identify common patterns and themes across the studies using the HEE capability framework as our initial thematic framework to code the data, whilst also using an inductive approach to allow for new themes and concepts to be detected.^13^ This involved reading the full texts and data extraction table in detail and applying codes to the data, which were grouped into themes. We then conducted a narrative synthesis, which involved summarizing the themes in a narrative summary. Our analytical focus was primarily on the competencies and educational interventions surrounding digital literacy education. This included the aim, structure, teaching methods, delivery modes, targeted skills, assessment strategies, and resultant outcomes of these interventions. Constructive alignment theory was further used to assess the presence of alignment of the taught material and assessment approaches within the reviewed studies, only including studies with detailed description of educational interventions.^17^ To ensure the integrity and reliability of our findings, themes and sub-themes were iteratively reviewed, refined, and validated through multiple rounds of discussions among the research team until a consensus was achieved.

## Results

### Descriptive summary of the included studies

**Figure 2.**
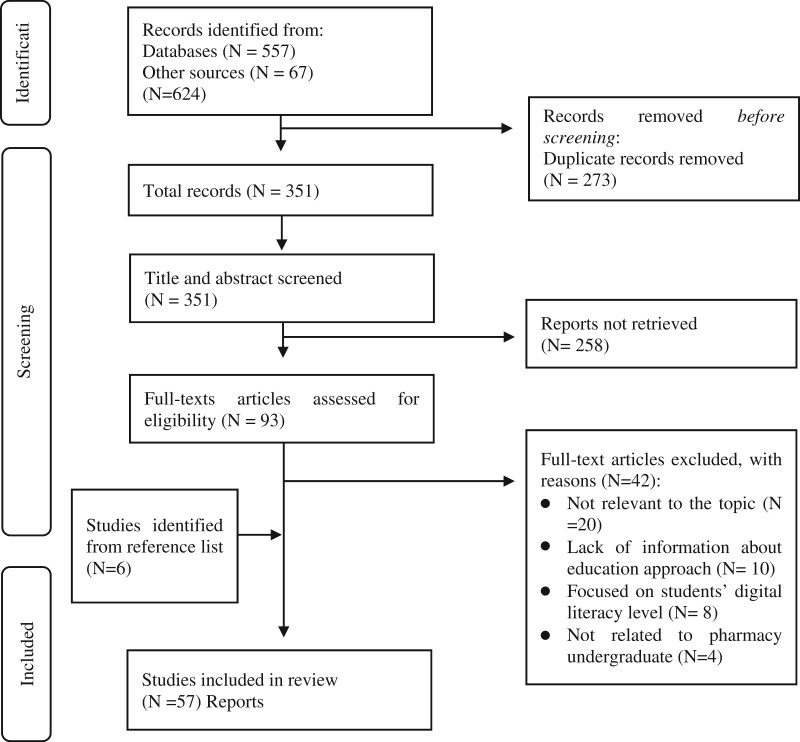
PRISMA flow diagram for the scoping review.

We identified 624 studies; 557 from database searches and 67 from other sources. Duplicates were removed (*n* = 273), leaving 351 studies for screening of title and abstract screening. We retrieved 93 studies for full-text review and 51 met the inclusion criteria; a further 6 were included through manual searching of reference lists. A total of 57 studies were included in this scoping review (Figure 2). Most of the studies (*n *= 36) were published between 2016 and 2022.^14^^,^^18–64^ The majority of the studies (*n* = 49) were conducted in the United States,^14^^,^^18–69^ with the other studies being conducted in Canada (*n* = 2),^29^^,^^70^ Romania (*n* = 1),^71^ Malaysia (*n* = 1),^72^ Singapore (*n* = 1),^73^ the United Kingdom (*n* = 1),^40^ Poland (*n* = 1),^48^ and 1 multi-country study (*n* = 1.).^14^ Out of the 57 studies, 11 focused on digital literacy competency and skills.^14^^,^^24–28^^,^^39^^,^^40^^,^^47^^,^^58^^,^^62^^,^^68^ The remaining studies covered various aspects of digital literacy education, including 23 studies on Electronic Health Record (EHR) skills,^22,30,32,33,^^35–38^^,41,42,^^44–46^^,^^49–51^^,54,55,60,^^63–65^^,73^ 14 studies on pharmacy informatics courses,^19,21,29,31,34,52,59,61,66,67,^^69–72^ 4 studies discussed tele-pharmacy,^20^^,^^43^^,^^48^^,^^53^ 3 studies on mobile health (mHealth),^18^^,^^23^^,^^27^ and 2 studies covered Prescription Drug Insurance tools.^56^^,^^57^ Additional details about the characteristics of the included studies can be found in Table S4. Table S5 provides an outline for the educational interventions, skills covered, and outcomes of each study.

**Figure 3.**
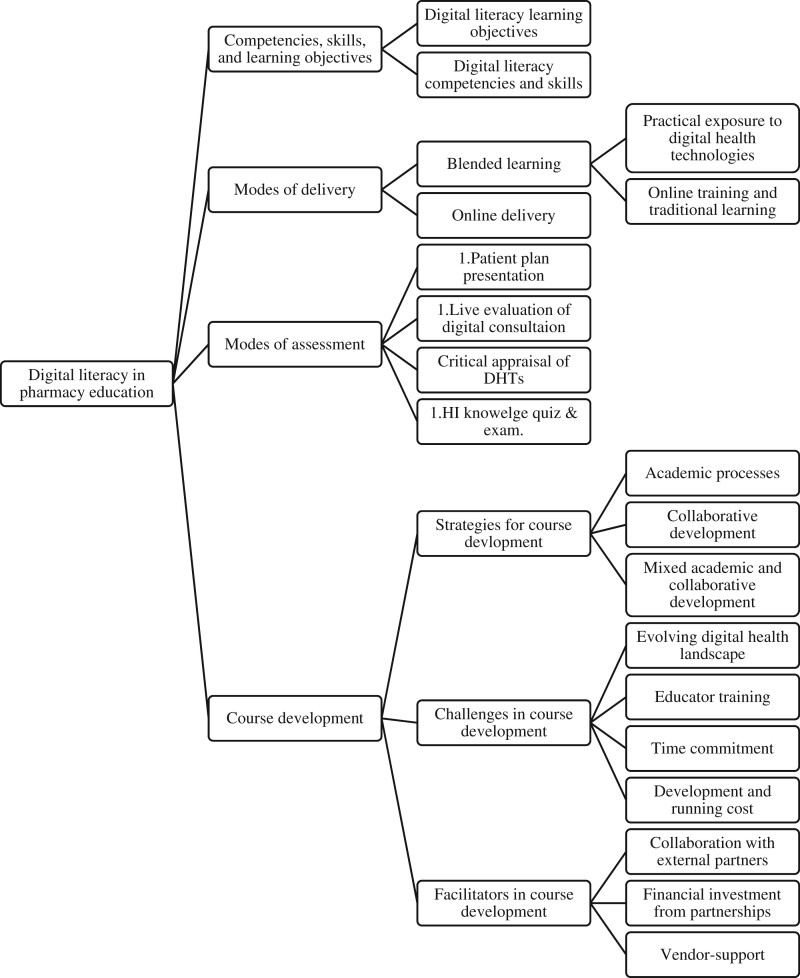
Thematic analysis of the digital literacy in pharmacy displaying the 4 themes and subthemes.

We identified 4 main themes, some of which had subthemes: (1) competencies, skills, and learning objectives, (2) mode of delivery, (3) assessment methods, and (4) course development (Figure 3). These themes, along with supporting evidence from relevant studies, are discussed in the following sections.

### Competencies, skills, and learning objectives

#### Digital literacy learning objectives

**Figure 4.**
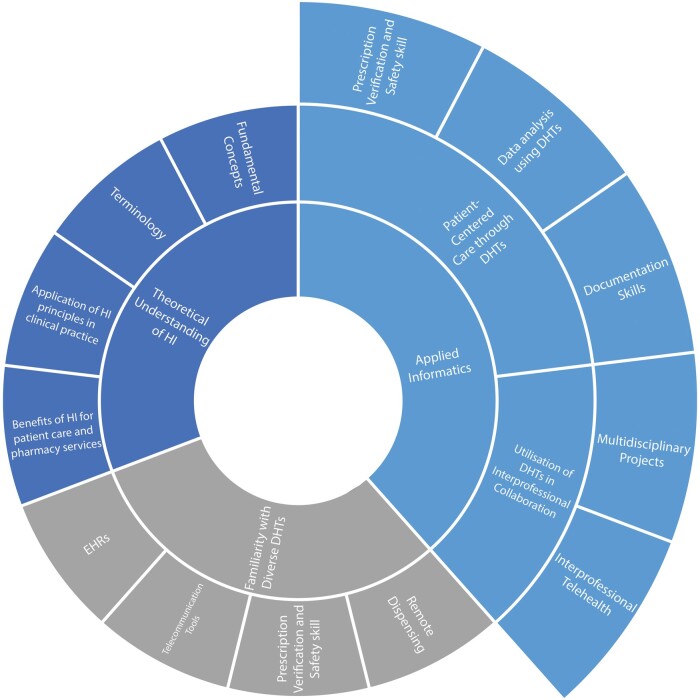
Learning objectives for incorporation of digital literacy in undergraduate pharmacy education.

The skills and learning objectives can be classified into 3 major categories: theoretical understanding of health informatics (HI), familiarity with diverse digital technologies, and applied informatics, which centered on 2 specific domains: the delivery of patient-centered care through DHTs and the utilization of DHTs in interprofessional collaboration (Figure 4).

Training aimed to provide students with a theoretical understanding of fundamental concepts and terminology in HI. Furthermore, the learning objectives included student understanding of the application of HI principles in clinical practice and the benefits of HI for patient care and pharmacy services.^21^^,^^69^^,^^71^^,^^72^ Moreover, the familiarization with various digital technologies intended to expose students to a broad range of technological tools employed in healthcare, thus enhancing their readiness to employ systems such as EHRs, automated technologies, telecommunication tools, and remote dispensing.^31^^,^^34^^,^^44^^,^^69^^,^^72^

Lastly, studies highlighted the application of informatics principles and DHTs in delivering patient-centered care and promoting interprofessional collaboration. This included enhancing documentation skills,^22^^,^^37^^,^^54^^,^^55^^,^^60^ analysis and interpretation of data using digital technology,^18^^,^^20^^,^^22^^,^^23^^,^^30^^,^^32^^,^^35^^,^^38^^,^^49^^,^^51^^,^^56^^,^^57^^,^^60^^,^^63^^,^^73^ and skills related to using digital systems for prescription verification, and medication safety monitoring.^41^^,^^42^^,^^49^ Interprofessional collaboration was developed through telehealth consultations, which required communication and collaboration within interprofessional teams for medication management.^56^^,^^57^ Multi-disciplinary projects involved developing an mHealth app prototype for smartphones that facilitated engagement with software engineers to address mobile technology requirements and find innovative solutions.^27^

#### Digital literacy competencies and skills

Numerous studies examined digital literacy competencies for pharmacy students, focusing on developing skills and knowledge related to informatics terminology, concepts, and building effective communication skills whilst using DHTs.^25^^,^^39^^,^^58^^,^^62^ Some studies specifically concentrated on competencies related to EHRs^40^^,^^47^ and mHealth.^68^

Most studies recommended the early introduction of training in undergraduate education, to provide a strong foundation in core competencies aligned with the ACPE standards.^25^^,^^39^^,^^58^^,^^62^ Fox et al^58^ proposed core competencies for pharmacy informatics education, including knowledge of basic terminology around technology, informatics, and healthcare; understanding reasons for the systematic processing of healthcare data; and recognizing the benefits and constraints of DHT, to develop practical informatics skills. Other approaches focused on introducing students to technologies used across each stage of the medication use process (ie, procurement, prescribing, order verification, compounding, dispensing, administration, and monitoring).^25^^,^^62^ Martin et al^39^ meanwhile outlined foundational and applied competencies in HI for pharmacy students. The foundational competencies covered HI concepts, ethical principles, health data management, project management, and communication skills among others. The applied competencies included incorporating HI into patient care, using health information technology for CDS systems, managing medication therapy, support population health management and supporting research.^39^

Studies also emphasized the importance of training pharmacy students on interoperability standards, biomedical informatics, emerging technologies, legal and regulatory aspects, patient outcomes and safety, and professional development.^25^^,^^39^^,^^62^ Flynn et al^26^ suggested that advanced informatics training should include domains like the design and modeling of information systems, development of prototypes, implementation of health IT, and evaluation of health information resources.

Some studies focused on providing students with opportunities to use and develop skills in using specific DHTs, such as EHRs and mHealth, to prepare them for real-world practice.^26^ In the United Kingdom, a National Working Group was formed to integrate EPRs into the undergraduate curriculum for healthcare students.^40^ They defined 6 domains of competence and 29 learning outcomes, including digital health work, accessing data, communicating, generating data, interdisciplinary collaboration, and monitoring and auditing.^40^

### Modes of delivery

#### Blended learning approach

The majority of the studies employed a blended learning approach.^18^^,^^20–23^^,27,^^30–35^^,37,38,^^41–44^^,46,48,49,51,^^53–57^^,60,61,^^63–65^^,^^70–73^ Blended learning approaches combined traditional face-to-face instruction with technology-mediated instruction, such as using digital technologies in the classroom,^20,22,23,27,^^30–32^^,34,35,37,38,^^41–44^^,46,48,49,51,53,54,56,60,61,^^63–65^^,^^70–73^ providing remote access to DHTs,^33^^,^^55^^,^^57^ and giving access to prerecorded online material.^18^^,^^21^^,^^42^^,^^74^

##### Practical exposure to digital health technologies

Wasynczuk and Sheehan^51^ integrated a teaching EHR system within a pharmacy school, giving students access to a simulated patient’s medication history and medical record. Students analyzed the data to produce an accurate response to a patient-specific medication query.^51^ Students’ perceptions of their knowledge and confidence in using EHR were reported to have improved (from 61.4% to 89.3% and 20.5% to 82.8%, respectively).^51^ Similarly, Neumann-Podczaska et al^48^ described an interprofessional telemedicine project, where pharmacy students engaged with medical students in teleconsultations and evidence-based recommendation. These authors reported increased student confidence and understanding of patient care post training.^48^ Three studies discussed remote access to DHT.^33^^,^^55^^,^^57^ Brown et al^55^ introduced an Internet-based medical chart in a pharmacotherapy course that students accessed from home. The web-based nature of the EHR enabled students to access patient information, develop clinical notes, and view progress notes remotely, however, the study reported connectivity and technical issues, which are important limitations to consider.^55^

##### Online training and e-learning

Online training material was used in some studies, alongside traditional learning methods.^18^^,^^21^^,^^42^ Hincapie et al^21^ integrated the online pharmacy informatics module “Partners in E (PinE),” covering fundamental concepts like, interoperability, data management, and DHT. A team-based learning (TBL) approach was used for preclass preparation, individual and team readiness assurance, problem-solving activities, and immediate feedback.^21^ Pre- and post-survey findings from 83 students revealed a considerable improvement in their knowledge and confidence.^21^

#### Online delivery approach

Learning content was delivered solely *via* online methods in 2 studies.^50^^,^^69^ One study used online modules covering digital literacy topics, including EHRs, telemedicine, and CDSSs.^69^ The online course significantly improved students’ knowledge, attitudes, and confidence in HI and student experience was positive.^69^ Another study integrated an EHR system into an online pharmacotherapy course where students practiced the pharmacist’s patient care process.^50^ The majority (60% [*n* = 15]) of students reported positive perceptions of this approach, recognizing its value in data collection, therapy assessment, plan development, monitoring/follow-up, and better preparation for experiential rotations involving EHR systems.^50^

### Modes of assessment

Strategies used to assess student proficiency and knowledge for the intended learning outcomes were grouped into 4 broad themes:

1. Presentation of a patient plan based on information retrieved from a digital system. This included several components such as completing Subjective, Objective, Assessment, and Plan (SOAP) notes,^22^^,^^37^^,^^38^^,^^54^^,^^55^^,^^60^^,^^64^ submitting handwritten progress notes of simulated cases,^33^^,^^35^^,^^41^^,^^63^ using EHRs to respond to patient-specific questions,^51^^,^^73^ presenting a simulated patient case to other students in a grand round format,^49^ and comparing prescription drug plans on an EHR system before choosing the most option.^56^^,^^57^
2. Live evaluations of digital consultation skills, for example, assessing student performance during telehealth activities.^53^
3. Critical appraisal of DHTs, where students completed a worksheet, and outlined their evaluation of different mHealth apps, to determine which one would be most effective for a specific healthcare need.^18^^,^^23^
4. Quiz and written exams of knowledge and understanding of fundamental HI concepts.^21^^,^^67^^,^^69^

Many of the included studies lacked a comprehensive and standardized description of their educational interventions, potentially affecting their effectiveness and reproducibility. However, in studies with detailed descriptions, constructive alignment theory was employed to link the learning objectives, educational activities, and assessments (see Table 2 and Table S6). Learning objectives were also mapped to the HEE capability framework to highlight areas that the education/training addresses.

**Table 2.** Displays the constructive alignment components that relate to the education and training and highlight the HEE domain which they focus upon.

HEE domain	Learning objective	Related teaching approach	Related assessment mode
Information, data, and content: able to gather and evaluate relevant health information and data using DHT.	Ability to gather and evaluate relevant patient information and data using EHRs.^22^^,^^33^^,^^35^^,^^37^^,^^41^^,^^42^^,^^46^^,^^49^^,^^51^^,^^54^^,^^55^^,^^60^^,^^73^	A hands-on approach to review simulated patient cases using EHRs and develop SOAP notes.^22^^,^^33^^,^^35^^,^^37^^,^^42^^,^^54^^,^^55^^,^^60^	Longitudinal case follow-up and SOAP note documentation.^22^^,^^37^^,^^54^^,^^55^^,^^60^ Completion of a handwritten progress note.^35^ Assessment of timing and quality of clinical decision-making for a simulated case with EHR access in the intervention group vs control group.^33^ Practical examination on medication orders and medication errors.^42^
		Incorporation of EHR systems in various courses.^46^^,^^49^	The study lacks clear information regarding the specific method used for assessment.^46^^,^^49^
		Case-based approach to use EHR for drug information inquiry.^51^	Written assignment: Gathering patient-specific information and writing a response to a drug-related question.^51^
		Simulated EHR using Mobile app.^71^^,^^73^	The study lacks clear information regarding the specific method used for assessment.^73^
	Ability to handle drug information.^72^	Practical sessions.^72^	Evaluation of pharmacy-related Internet sites and establishment of websites.^72^
	Ability to gather, evaluate patients’ information using telehealth.^20^	Simulated IP telehealth visit.^20^	The study lacks clear information regarding the specific method used for assessment.^20^
	Ability to find, evaluate, and effectively use mHealth technologies.^18^^,^^23^	Lectures cover topics on mHealth.^18^^,^^23^ Workshop focused on evaluation of mHealth.^18^^,^^23^	Worksheet for students to evaluate specific mHealth devices.^23^ Evaluate medical apps using the evaluation tool and presented their findings to the class.^18^
	Ability to evaluate patients’ information using Medicare Part D plans.^56^^,^^57^	Lectures on the Medicare Part D program and its basic concepts.^56^^,^^57^	Group project where students develop a list of “The Top 10 Things Every Medicare Beneficiary Should Know about Medicare Part D and presents it to the class.^56^ The study lacks clear information regarding the specific method used for assessment.^57^
Communication, collaboration, and participation: Able to effectively communicate and collaborate with other healthcare professionals using digital platforms and tools to improve patient outcomes.	Ability to discuss health technologies with other health care professionals for the common benefit of safe, quality patient care.^69^	Online course on HI.^69^	Participation in online discussions.^69^
	Ability to provide healthcare services to rural area through telehealth.^53^	Combination of didactic coursework and experiential learning in tele pharmacy.^53^	Quality of documentation of a daily log sheet of their activity.^53^ Live evaluations of student performance in telecommunication stations by the faculty mentor.^53^
	Ability to communicate and collaborate effectively as an IP team.^20^^,^^48^	Simulated IP telehealth visit.^20^^,^^48^	The study lacks clear information regarding the specific method used for assessment.^20^^,^^48^
	Ability to communicate mobile technology needs and solutions with software engineers.^27^	Course in mHealth for pharmacy and computer science students.^27^	Worked in teams to complete various assignments, including: Develop a course proposal and presented 30-min “pitch” presentations on proposed courses,^27^ and developed purchase plans for mHealth lab equipment.^27^
Technical proficiency: Able to navigate and troubleshoot digital health tools and software with confidence and efficiency.	Proficiency in using HI systems and software and accessing drug information sources.^70–72^	Hands-on experience with software applications in pharmacy.^71^	The study lacks clear information regarding the specific method used for assessment.^71^
		Practical session on drug information handling skills.^72^	Evaluation of pharmacy-related Internet sites and the establishment of websites.^72^
		Incorporation of hospital information system in HI course.^70^	Practical exams on clinical workflows: Complete clinical and administrative tasks related to pharmacy management.^70^
	Ability to navigate and use of EHRs.^22^^,^^33^^,^^35^^,^^37^^,^^38^^,^^41^^,^^42^^,^^46^^,^^49^^,^^51^^,^^54^^,^^55^^,^^60^^,^^63^^,^^73^	Simulated EHR experience.^22^^,^^33^^,^^35^^,^^37^^,^^42^^,^^54^^,^^55^^,^^60^^,^^63^	Longitudinal case follow-up and SOAP note documentation.^22^^,^^37^^,^^54^^,^^55^^,^^60^ Completion of a handwritten progress note.^35^ Assessment of timing and quality of clinical decision-making for a simulated case with EHR access in the intervention group versus control group.^33^ Complete of patient-centered tasks including medication reconciliation, compilation of patient information, identification and resolution of drug therapy problems, verification of sterile products, and presentation of patient cases.^63^ Practical examination on medication orders and medication errors.^42^
		Simulated EHR using Microsoft PowerPoint slides.^38^	The studies lack clear information regarding the specific method used for assessment.^38^^,^^49^^,^^73^
		Simulated EHR using mobile app.^73^	
		Incorporation of EHR systems in various courses.^49^	
		Case-based approach to use EHR for drug information inquiry.^51^	Assignment: Gathering patient-specific information and writing a response to a drug-related question.^51^
		Remote accessing and reviewing EHR of real patients.^41^	A competency-based assessment to evaluate pharmacy students’ medication safety skills.^41^
		EHR training approaches in classroom and during APPEs.^46^	The study lacks clear information regarding the specific method used for assessment.^46^
	Ability to use online Medicare tools in evaluating and enrolling in Medicare Part D plans using online tools.^56^^,^^57^	Introducing a teaching module on Medicare prescription drug.^56^	Written assignment: Comparing a 3 Medicare plans and justifying their choice.^56^ Mock counseling activity.^56^
		A PBL session using Medicare prescription drug plan finding tool.^57^	Case-based exercise to identify the 3 least expensive plans and corresponding costs.^57^
	Ability to navigate and use telehealth tools.^20^^,^^48^^,^^53^	Combination of didactic coursework and experiential learning in tele pharmacy.^53^	Evaluation of student performance: Quality of documentation of a daily log sheet of their activity.^53^ Live evaluations of student performance in telecommunication stations by the faculty mentor.^53^
		Simulated IP telehealth visit.^20^^,^^48^	The study lacks clear information regarding the specific method used for assessment.^20^^,^^48^
Creation, innovation, and research: Able to create and innovate digital health solutions to improve patient care and outcomes and conduct research using DHT.	Ability to design and implement mHealth interventions.^27^	Project-based learning and PBL was used mHealth course.^27^	mHealth project: Developed purchase plans for mHealth lab equipment.^27^ Development of an mHealth application.^27^
Objectives not covered by the HEE capabilities framework.	Demonstrate understanding of basic concepts of HI technology.^21^^,^^34^^,^^57^^,^^72^	Introducing a pharmacy informatics course using TBL approach.^21^	Individual readiness assurance test, team readiness assurance test, and application exercises to evaluate and analyze case scenarios related to HIT and informatics encountered in pharmacy.^21^
		Implementation of new course “Pharmacoinformatic” involving lectures on drug information basics.^72^	Coursework (40%) essay final examinations (60%).^72^
		Online lectures on health technologies and informatics integration in pharmacy.^69^	Multiple-choice, true-false, and short-answer questions administered at regular intervals.^69^
		Facilitated discussion on pharmacist’s role in pharmaceutical care.^63^	Short quiz assessing understanding of concepts related to readings and laboratory activities.^63^
		Lectures on a specific digital technology for example, Medicare Part D.^57^	Case-based exercise to identify the 3 least expensive plans and corresponding costs.^57^
		Informatics skills laboratory, covering DHT such as types of automation equipment and the role of technology in the medication use process.^34^	Studies lacks clear information regarding the specific method used for assessment.^34^

### Course development

#### Strategies for developing courses

Various strategies have been utilized in developing digital health courses at undergraduate pharmacy schools. These approaches include academic processes such as literature reviews,^69^ evaluations of existing courses,^29^^,^^71^ and course piloting.^34^^,^^49^ Also, collaborative working with organizations,^29^^,^^66^ experts,^53^^,^^57^ commercial companies,^31^^,^^34^ and other schools.^18^^,^^20^

Fuji et al^69^ developed an HI elective course based on a literature review with topics selected through an evidence-based approach, stakeholder suggestions, and reviewing informatics in pharmacy practice. Rocchi et al,^29^ updated an existing e-resource prepared by the Association of Colleges of Pharmacy in Canada, addressing gaps in informatics content in undergraduate curricula.^29^ Rigorous editorial advisory group and peer reviews were undertaken to ensure the resource was relevant and up-to-date.

Key expert stakeholder involvement was important in several studies, that obtained feedback from clinicians, faculty members, and health policy experts on the developed courses.^53^^,^^57^ Seifert et al^53^ reported developing a tele-pharmacy teaching model through discussions with members from the Office of Rural and Community Health and the Texas Tech University Health Sciences Centre Telemedicine program, as well as a rural telehealth practitioner. This allowed the team to leverage prior telemedicine experience and ensured compliance with accreditation bodies and regulatory compliance organizations.^53^ Darley and Logan reported collaborating with a business technology company to provide pharmacy students with a course on automation technology. Company representatives demonstrated their automation equipment on-site, and the course focused on the role of technology in medication usage.^34^ This approach resulted in significant increases in student confidence and understanding of informatics technologies.^34^

Lastly, some pharmacy schools collaborated to co-develop a course.^18^^,^^20^ Rodis et al^18^ reported a collaboration between Ohio State University and Massachusetts College of Pharmacy schools to develop an innovative learning experience for pharmacy students centered on identifying, reviewing, and using medical apps. The course successfully improved perceptions of student skills (*n* = 119), in the following areas: finding (44% vs 95%), evaluating (15% vs 93%), and using medical applications in patient care (26% vs 90%).^18^

#### Challenges and facilitators in course development

Several challenges to incorporating digital health into pharmacy education have been recognized, including the ongoing evolution of digital health, educator training, and the expense and time required to develop such courses.^20,21,23,25,37,^^69–72^ The broad scope of HI, and the field’s constant innovation, make it challenging to maintain course content.^69^^,^^71^ There is also a lack of skilled professionals with the necessary knowledge to educate others.^20^^,^^25^^,^^70^ The cost of DHTs, and the number of devices required, were further challenges, along with the need to frequently update the equipment to meet modern standards.^20^^,^^23^ Additionally, some pharmacy schools reported hesitation in investing resources in new courses without evidence of their effectiveness.^21^^,^^37^^,^^72^

On the other hand, numerous research has identified factors that encourage the development of digital health education and training.^21^^,^^22^^,^^31^^,^^34^^,^^55^^,^^63^^,^^66^ Collaboration with external partners, such as DHT businesses may also be financially advantageous. In 2 studies, DHT providers gave system demos for free, as a part of community service.^31^^,^^34^ One university also received support from an EHR provider—MEDITECH software package—to implement the system across all health professions programs.^63^

## Discussion

### Principal findings

This is the first study to focus on incorporating digital literacy education into undergraduate pharmacy programs. The scoping review provides a comprehensive analysis of the existing literature across multiple countries, identifying 57 relevant studies that present strategies for educating, training, and assessing digital literacy in pharmacy students and trainees. The educational approaches presented in these studies focused on various areas, including theoretical understanding of HI, familiarity with digital technologies, patient-centered care through digital technologies, and interprofessional collaboration using digital technologies. A range of delivery and assessment approaches were also identified, with blended approaches being the most utilized. The studies showed a diverse range of approaches to course development. Our analysis suggests that external engagement with practitioners and/or industrial partners can enhance the successful implementation of digital literacy education in undergraduate pharmacy programs. These collaborations provide access to real-world platforms, DHTs, and the opportunity to interact with industry professionals.^34^ However, there is potential for conflict of interest with third parties which can undermine the integrity and quality of the educational course. Following policies and guidelines to ensure sustainable and ethical collaborations is therefore vital.^75–77^ Our review also identified foundational skills in pharmacy undergraduate education, including fundamental concepts related to HI, such as terminology, basic knowledge, ethical principles, health data management, and communication skills. Exposure to commonly used pharmacy technologies, such as EHRs, mHealth, and telehealth was considered important. Additionally, advanced skills encompassed domains like interoperability standards, biomedical informatics, information system design and modeling, and emerging technologies (eg, digital medicine, genomics, AI, and robotics).

The findings align with previous research in medical and nursing education, which emphasizes a growing recognition of the digital health role.^78–80^ Healthcare professionals, including pharmacists, are expected to use DHT in a patient-centered manner. This involves learning how to effectively engage patients in the use of technologies and as a communication tool.^81^  In a comprehensive study by Zainal et al.^78^ several essential components of a clinical informatics (CI) curriculum for medical schools were identified, including CI utilization in clinical practice, ethical implications, CI-key concepts, and digital health. They also reported similar modes of delivery, including lectures, problem-based learning (PBL), and e-learning.^78^ Notably, they emphasized the importance of incorporating ethical principles when utilizing machine learning and AI tools, as well as understanding the potential limitations and biases of technology. This is essential to ensure a balance between technological advancements and compassionate patient care that ensures ethical and humanistic principles are upheld. Harerimana et al^79^ noted a lack of certainty around how to integrate HI concepts into undergraduate nursing education in Australia. However, utilizing DHTs within nursing education was reported in various studies, as they play a pivotal role in supporting teaching and learning using different formats, including face-to-face, online, and blended learning, during both classroom and simulation sessions.^79^

Our study assessed the alignment of learning objectives with the HEE capability framework for digital literacy in pharmacy education. While the framework provided useful guidance, we identified certain gaps in the current teaching practices. Specifically, there was limited emphasis on the development of skills related to creating and innovating digital health solutions to improve patient care and outcomes, as well as conducting research using DHT. Additionally, the importance of interdisciplinary collaboration in digital literacy education and the need to create appropriate digital identities in professional and personal contexts were often overlooked. These findings indicate potential areas for future development and improvement in digital literacy education within undergraduate pharmacy programs.

Different approaches to introducing HI into the educational curriculum were explored in our study. While there exists no consensus on the best approach, O’Connor and LaRue proposed a spiral approach of gradually and iteratively introducing new concepts to students.^82^ Further work is needed to explore the effectiveness of this.^82^ Hare et al,^83^ meanwhile suggested a tiered informatics curriculum design for medical education, which included different levels of depth within courses that students could opt into, ranging from shorter crash courses to longer fellowships based on their preferences. Successful implementation, however, depends on careful planning, resource allocation, and collaboration with healthcare institutions.^83^

The presence of educators with informatics expertise is critical for effective digital literacy education. Our study findings indicate that a lack of skilled informatics professionals has been identified as a significant barrier in the development of digital literacy courses.^20^^,^^25^^,^^70^ To address this challenge, informatics experts who are actively engaged in the field should be involved as educators, to enhance digital literacy education for aspiring healthcare professionals. It will therefore be necessary to provide support and resources to these educators, for example, dedicated time for education and establishing clear pathways for their continued informatics learning.

### Recommendation

The review provides recommendations for incorporating digital literacy education into undergraduate pharmacy programs. Table 3 presents specific recommendations to guide pharmacy schools in curricula development. These recommendations include emphasizing foundational knowledge, educators keeping up with emerging technology, recognizing the fast-paced nature of DHT, using standardized guidelines for reporting educational interventions to allow better sharing of good practice and harnessing partnerships with stakeholders to co-develop digital literacy education.

**Table 3.** Suggested recommendations for incorporating digital literacy education into undergraduate pharmacy programs.

Key recommendation	Specific recommendations
Acknowledge the rapidly evolving landscape of DHT: DHT is constantly evolving, and healthcare professionals must be able to adapt and stay up to date with new developments to provide the best possible care.	Emphasize foundational knowledge by providing students with a basic foundation in digital literacy to adapt to new DHTs such as digital medicine, genomics, artificial intelligent, and robotics. Identify advanced competencies which can be offered in a postgraduate or workforce training. Educators to keep up to date with emerging technology and explore how best to incorporate into the curriculum, for example, via continuous professional development, membership of workforce development or specialist interest, groups
Report educational interventions in a standardized approach: Standardized reporting ensures that interventions are adequately described, evaluated, replicated, and applied in different contexts, allowing for better assessments of effectiveness and reproducibility.	Use of standardized reporting guidelines such as template for intervention description and replication (TIDieR) and the guideline for reporting evidence-based practice educational interventions and teaching (GREET) to report digital health interventions in a consistent and transparent manner.^84^^,^^85^
Ensure constructive alignment between learning objectives, educational interventions, and assessment: Constructive alignment ensures that the interventions and assessments are appropriate for developing necessary knowledge and skills and that the assessment is an appropriate measurement of that knowledge and skills.	Ensure that the learning objectives align with the necessary knowledge and skills for developing digital health literacy. Develop educational interventions that are designed to achieve these learning objectives. Design assessments that appropriately align to the objectives and educational experience.
Partnership with stakeholders to facilitate development of digital literacy courses: Collaboration with stakeholders, including other educational institutions and digital health companies, facilitates student access to the necessary resources to develop their digital literacy skills and stay up to date with the latest DHTs.	Collaborate with other educational institutions, practitioners, and industry partners to provide access to necessary skills, expertise, and resources. Establish partnerships with health technology vendors to facilitate training and support for digital health technologies.

### Limitations

Although a comprehensive search of major bibliographic databases was conducted, it is possible that some relevant sources, such as unpublished studies or articles in the gray literature, may have been missed. Additionally, some reviewed studies lacked detailed descriptions of digital health courses design and implementation, including learning objectives, development processes, and assessment approaches, which limit the depth of findings. It is also important to note that interrater reliability between reviewers was not assessed, which might have led to some discrepancies in how the included studies are interpreted and evaluated. Finally, as the majority of the studies were conducted in the United States and primarily in English, the applicability of specific learning approaches in an international context may differ due to differences in healthcare and educational systems.

## Conclusions

In summary, this scoping review highlights the importance of integrating digital literacy education into undergraduate pharmacy curricula. It identifies learning objectives, skills, competencies, teaching, and assessment methods to achieve this goal. Given the rapid advancement of DHT, healthcare professionals and educators must remain up to date with new developments to provide optimal patient care and appropriate education. We have also identified several recommendations, including prioritizing foundational knowledge, identifying advanced digital literacy competencies, keeping pace with emerging technology, using standardized guidelines for reporting interventions, and partnering with stakeholders to facilitate the development of digital literacy courses. Despite limitations, this study offers a valuable overview of the current state of digital literacy education in undergraduate pharmacy programs and practical suggestions for enhancing digital literacy education to equip future pharmacists for the changing landscape of DHT.

## Supplementary Material

ocad223_Supplementary_Data
