# Supplementary material for: Digital literacy in undergraduate pharmacy education: a scoping review
Source: J Am Med Inform Assoc. 2023 Dec 5;31(3):732–45. doi: 10.1093/jamia/ocad223 (PMC10873839; doi:10.1093/jamia/ocad223)
Supplement: ocad223_Supplementary_Data [file ocad223_supplementary_data.docx]

**Supplemental Table 1. Search strategy**

Database:
Ovid MEDLINE(R) and In-Process, In-Data-Review & Other Non-Indexed Citations <1946 to June 17, 2022>

| # | Query | Results from 17 Jun 2022 |
| --- | --- | --- |
| 1 | Students, Pharmacy/ | 4133 |
| 2 | Pharmacy undergraduate*.mp. | 88 |
| 3 | MPharm.mp. | 45 |
| 4 | PharmD.mp. | 835 |
| 5 | Foundation trainees.mp. | 60 |
| 6 | Pre Registration pharmacy.mp. | 4 |
| 7 | Pharmacy Intern.mp. | 29 |
| 8 | Doctor of pharmacy.mp. | 811 |
| 9 | Master of pharmacy.mp. | 51 |
| 10 | 1 or 2 or 3 or 4 or 5 or 6 or 7 or 8 or 9 | 4940 |
| 11 | Computer Literacy/ or Internet/ or Digital literacy.mp. or Telemedicine/ | 111392 |
| 12 | Electronic Health Records/ or Digital health.mp. | 30119 |
| 13 | Health informatics.mp. or Medical Informatics/ | 16056 |
| 14 | E-health.mp. | 3585 |
| 15 | Electronic health.mp. or Medical Records Systems, Computerized/ | 57138 |
| 16 | 11 or 12 or 13 or 14 or 15 | 179772 |
| 17 | Education, Pharmacy/ or Education/ | 28533 |
| 18 | Knowledge/ | 13317 |
| 19 | skill*.mp. | 255046 |
| 20 | Teaching/ | 51304 |
| 21 | Learning/ | 75446 |
| 22 | Curriculum/ | 83343 |
| 23 | courses.mp. | 81112 |
| 24 | syllabus.mp. | 962 |
| 25 | syllabuses.mp. | 73 |
| 26 | Programme.mp. | 107906 |
| 27 | competency.mp. | 40902 |
| 28 | competencies.mp. | 18903 |
| 29 | Standard.mp. | 1002912 |
| 30 | 17 or 18 or 19 or 20 or 21 or 22 or 23 or 24 or 25 or 26 or 27 or 28 or 29 | 1622084 |
| 31 | Training.mp. | 518964 |
| 32 | Practice.mp. | 1150039 |
| 33 | Experiential.mp. | 10676 |
| 34 | 31 or 32 or 33 | 1581307 |
| 35 | Assessing.mp. | 307419 |
| 36 | assess*.mp. | 3783290 |
| 37 | Evaluat*.mp. | 4373100 |
| 38 | examination.mp. | 796294 |
| 39 | examine.mp. | 693441 |
| 40 | Rate.mp. | 2588571 |
| 41 | 35 or 36 or 37 or 38 or 39 or 40 | 9486998 |
| 42 | 30 or 34 or 41 | 10992861 |
| 43 | 10 and 16 and 42 | 193 |

**Supplemental Table 2: Detailed Search Strategy ( June, 2022)**

| **PubMed** | ("Students, Pharmacy"[mesh] OR "Pharmacy undergraduate* "[tiab] OR "MPharm "[tiab] OR "Master of Pharmacy "[tiab] OR "PharmD"[tiab] OR "Doctor of pharmacy "[tiab] OR "foundation train* Pharmacy "[tiab] OR "Pre-register* pharmacy "[tiab] OR "Pharmacy Intern"[tiab] ) AND ("Medical Informatics"[mesh] OR "Telemedicine"[mesh] OR "Computer Literacy"[mesh] OR "Digital health"[tiab] OR "digital literacy"[tiab] OR "health informatics"[tiab] OR "E-health"[tiab] OR "electronic health "[tiab]) AND ("Curriculum"[mesh] OR "Education"[mesh] OR "Knowledge"[mesh] OR "Teaching"[mesh] OR "Learning"[mesh] OR "Skills"[tiab] OR "competency"[tiab] OR "competencies"[tiab] OR "syllabus"[tiab] OR "training"[tiab] OR "Practice"[tiab] OR "experiential"[tiab] OR "assess*"[tiab] OR "evaluat*"[tiab] OR "examin*"[tiab] OR "Rate"[tiab]) |
| --- | --- |
| **Embase** | (exp Students, Pharmacy/ OR Pharmacy undergraduate* .mp. OR MPharm .mp. OR Master of Pharmacy .mp. OR PharmD.mp. OR Doctor of pharmacy .mp. OR foundation train* Pharmacy .mp. OR Pre-register* pharmacy .mp. OR Pharmacy Intern.mp. ) AND (exp Medical Informatics/ OR exp Telemedicine/ OR exp Computer Literacy/ OR Digital health.mp. OR digital literacy.mp. OR health informatics.mp. OR E-health.mp. OR electronic health .mp.) AND (exp Curriculum/ OR exp Education/ OR exp Knowledge/ OR exp Teaching/ OR exp Learning/ OR Skills.mp. OR comptencey.mp. OR comptencies.mp. OR syllabus.mp. OR training.mp. OR Practice.mp. OR experiential.mp. OR assess*.mp. OR evaluat*.mp. OR examin*.mp. OR Rate.mp.) |
| **Scopus** | ( TITLE-ABS-KEY ( "pharmacy student" ) OR TITLE-ABS-KEY ( "Pharmacy undergraduate" ) OR TITLE-ABS-KEY ( "MPharm " ) OR TITLE-ABS-KEY ( "Master of Pharmacy " ) OR TITLE-ABS-KEY ( "PharmD" ) OR TITLE-ABS-KEY ( "Doctor of pharmacy " ) OR TITLE-ABS-KEY ( "foundation trainee Pharmacy " ) OR TITLE-ABS-KEY ( "Pre-registration pharmacy " ) OR TITLE-ABS-KEY ( "Pharmacy Intern" ) ) AND ( TITLE-ABS-KEY ( "Computer Literacy" ) OR TITLE-ABS-KEY ( "Medical Informatics" ) OR TITLE-ABS-KEY ( "Digital Health" ) OR TITLE-ABS-KEY ( "Telehealth" ) OR TITLE-ABS-KEY ( "Telemedicine" ) OR TITLE-ABS-KEY ( "Health Informatics" ) OR TITLE-ABS-KEY ( "digital literacy" ) OR TITLE-ABS-KEY ( "E-health" ) OR TITLE-ABS-KEY ( "electronic health " ) OR TITLE-ABS-KEY ( "electronic health " ) ) AND ( TITLE-ABS-KEY ( "Curriculum" ) OR TITLE-ABS-KEY ( "Education" ) OR TITLE-ABS-KEY ( "Knowledge" ) OR TITLE-ABS-KEY ( "Teaching" ) OR TITLE-ABS-KEY ( "Learning" ) OR TITLE-ABS-KEY ( "Skills" ) OR TITLE-ABS-KEY ( "competency" ) OR TITLE-ABS-KEY ( "competencies" ) OR TITLE-ABS-KEY ( "syllabus" ) OR TITLE-ABS-KEY ( "training" ) OR TITLE-ABS-KEY ( "Practice" ) OR TITLE-ABS-KEY ( "experiential" ) OR TITLE-ABS-KEY ( "assess" ) OR TITLE-ABS-KEY ( "evaluate" ) OR TITLE-ABS-KEY ( "examine" ) OR TITLE-ABS-KEY ( "Rate" ) ) |
| **CINAHL** | ((MH "Students, Pharmacy+") OR AB("Pharmacy undergraduate* ") OR TI("Pharmacy undergraduate* ") OR AB("MPharm ") OR TI("MPharm ") OR AB("Master of Pharmacy ") OR TI("Master of Pharmacy ") OR AB("PharmD") OR TI("PharmD") OR AB("Doctor of pharmacy ") OR TI("Doctor of pharmacy ") OR AB("foundation trainee Pharmacy ") OR TI("foundation trainee Pharmacy ") OR AB("Pre-registeration pharmacy ") OR TI("Pre-registeration pharmacy ") OR AB("Pharmacy Intern") OR TI("Pharmacy Intern") ) AND ((MH "Computer LiteracyScope+") OR (MH "Medical Informatics+") OR (MH "Digital Health+") OR (MH "Telehealth+") OR (MH "Telemedicine+") OR (MH "Health Informatics+") OR AB("digital literacy") OR TI("digital literacy") OR AB("electronic health ") OR TI("electronic health ") OR AB("E-health") OR TI("E-health")) AND ((MH "Teaching Methods, Clinical+") OR (MH "Teaching Materials+") OR (MH "Learning+") OR (MH "Learning Methods+") OR (MH "Experiential Learning+") OR (MH "Knowledge+") OR (MH "Education+") OR (MH "Curriculum+") OR AB("competency") OR TI("competency") OR AB("competencies") OR TI("competencies") OR AB("syllabus") OR TI("syllabus") OR AB("training") OR TI("training") OR AB("Practice") OR TI("Practice") OR AB("experiential") OR TI("experiential") OR AB("assess") OR TI("assess") OR AB("evaluate") OR TI("evaluate") OR AB("examine") OR TI("examine") OR AB("Rate") OR TI("Rate")) |

**Supplemental Table 3.: Data extraction instrument**

| **Study id (first author and year of publication)** | **Title of the article** | **Country** | **Study design/type of study** | **Study Population** | **Objectives** | **Digital health focus** | **Details about course/ activity structure** | **Course development process** | **Learning objectives** | **Course format** | **Mode of delivery** | **Teaching approach** | **Course content** | **Assessment used in the course/curriculum,** | **Course evaluation** | **Facilitators/barriers related to digital literacy courses** | **Authors conclusion** | **Notes/additional information** |
| --- | --- | --- | --- | --- | --- | --- | --- | --- | --- | --- | --- | --- | --- | --- | --- | --- | --- | --- |
|  |  |  |  |  |  |  |  |  |  |  |  |  |  |  |  |  |  |  |

**Supplemental Table 4. Summary of basic characteristics of the included studies**

| **Study ID (first author and year of publication), Country.** | **Participants, Institution(s)** | **Digital health area and course content** | **Modality (in-person, online, blended course); Type of course (elective, core, integrated, standalone)** | **Teaching approach (lectures, lab-based); Teaching support tools** | **Course Development approach and frameworks used** | **Mode of Assessment** | **Evaluation of Students’ performance** | **Evaluation of the Course** |
| --- | --- | --- | --- | --- | --- | --- | --- | --- |
| Mihalas 1997(70), Romania, | Pharmacy students in Timisoara, Romania | Digital literacy course/curricula  The primary focus of the course was to provide a foundation in health informatics, including the role of pharmacists in this field, the fundamental concepts of health informatics, the use of technologies in pharmacy, and basic computer skills and documentation proficiency. | Blended (using software)  Core, standalone as a pharmaco-informatics course. | Lectures and lab-based approach | Course Development approach: updating an existence course used in medical school  Frameworks used: NA | NA | NA | NA |
| Ab Rahman 2002(71),  Malaysia, | Fourth-year students of pharmacy | Digital literacy course/curricula  The aim of the course was to mitigate pharmacists' potential fear or discomfort with computers and equip students with the essential knowledge and competencies required to manage drug information effectively. It also intended to educate students on the role of computer-assisted information management in the healthcare system. | In-person course.  Core course, standalone as a pharmaco-informatics course. | Lectures | Course Development approach: NA  Frameworks used: NA | Coursework and final exams with essays | knowledge:   - Using Questionnaire - Most students stated that their knowledge increased after the course. | Students’ opinion:   - Using Questionnaire - Many students found the course irrelevance to pharmacy practice Corse - some students found the course not well conducted, and its contents were in sufficient - Students were satisfied with the coursework. |
| Seifert 2002(52),  USA | Third and fourth year of PharmD students | Tele-pharmacy  A tele-video-based system of remote dispensing services was implemented to provide tele-pharmacy services in rural areas, allowing students to participate in the delivery of these services. | Blended (In person using online technology).  Core, integrated into a rural clerkship | Lectures and a “hands on” experience. | Course Development approach: Expert Consultation  Frameworks used: NA | live evaluations of student performance, and an assessment of the documentation’s quality. | Documentation:   - Analysis of the activity. - most of their activities were poorly documented | Experience:   - Using survey - didactic courses were excellent (2.82/3.0) - clerkship evaluations were fair to good (2.64–3.62/5.0) - 75% recommending the site to future students. |
| Brown 2005 (53),  USA, | Second year PharmD students | Electronic Health Records (EHRs)  A web-based platform known as the Internet-based Medical Chart (IMC) was created to facilitate the documentation of simulated patient care follow-up activities. This platform incorporated live actors who played the role of patients. | Blended (In person using online technologies).  Core, Integrated | Simulated case-based | Course Development approach: NA  Frameworks used: NA | Completion of SOAP note | Performance:   - Analysis of the activity - students' performance identified challenging areas, including incomplete documentation of therapeutic goals and specific follow-up laboratory test recommendations. | End-of-course evaluation:   - Compared to paper- based system, students prefer using EHR 95.5%. - 91% reported ease of use. |
| Zagar 2007 (55),  USA, | Second and third years of PharmD students. | Prescription Drug Insurance  The educational experience aimed to offer students a practical learning opportunity to utilize the web-based resources accessible to Medicare beneficiaries. It allowed them to assess drug plan options and hone their counseling skills while dealing with elderly patients who often have multiple unanswered queries. The program emphasized providing a realistic setting to prepare students for future professional encounters and improve their understanding of the healthcare needs of elderly patients. | Blended (In person using web-based tool)  Elective, Standalone course ‘‘Medicare 2006: This Year, It’s Different!’’ | lectures, mock counselling sessions  Medicare Part D Tool | Course Development approach: NA  Frameworks used: NA | Individual Assignment, select an appropriate Medicare drug plan for the given drugs. | Performance:   - Analysis of the activity, Based on completion of exercise. - average scores on the individual Top 10 lists, mock counselling sessions, and individual Medicare plan selection assignments all at or above 90%. | course evaluation:   - using survey - Students’ responses were uniformly positive, with the scores ranging from 4.6 to 4.8., |
| Brown 2007 (54),  USA, | Third year pharmacy students | Electronic Health Records (EHRs)  The curriculum was enhanced by the introduction of an Electronic Health Record (EHR) system and the addition of four "finish the SOAP note" activities, which aimed to provide students with practical experience in using the EHR system for documentation of patient care.  . | Blended course, (EHR can be accessed online off-campus).  Core, Integrated | Case-based learning a part of classroom lectures. | Course Development approach: NA  Frameworks used: NA | Individual assignment, Completion of SOAP note using Internet- based EHR | Performance:   - Analysis of the activity - 86.3% reported improved their pharmacotherapy knowledge - 78.2% reported improvement on documentation skills - 87% of the students avoided repeating previous mistakes by their final documentation activity. | End-of course   - Using Survey, - lowest ratings came on the timeliness of and usefulness of feedback, with 46.0% and 43.5%.   Evaluators’ opinion:   - Overall agreements that system easy to use - felt the exercises fostered improvement in both pharmacotherapy knowledge and documentation skills. |
| Cutler 2008 (56),  USA, | Second year pharmacy students | Prescription Drug Insurance  A Medicare Part D finder tool was incorporated into the "Health Policy for Pharmacists" course, aiming to introduce students to the organization, financing, and delivery of healthcare in the United States. The tool allowed students to explore and evaluate various drug plan options available to Medicare beneficiaries, enhancing their understanding of healthcare policy and its impact on patient care. | Blended (In person using web-based tool),  Core, Integrated | Lectures, problem-based learning | Course Development approach: Expert consultations  Frameworks used: NA | NA | knowledge, skill proficiency and attitudes:   - Pre- and post-tests evaluate students’ change - significant improvement was seen on all general knowledge questions about Medicare Part D, the greatest improvement was for the question on Medicare Plan Finder use, correct responses rose from 23.7% to 83.3%. - significant improvement in confidence from changed 2.9(1.3) to 4.3(0.6) - Students’ measured attitude did not change in general except for question related to “Pharmacists serve as patient advocates” which increased significantly 3.8 (0.9) to 4.5 (0.7) | NA |
| Fox 2008 (57),  USA | Pharmacy schools in USA | Recommendations for educating digital literacy  The objective of the study was to analyse the existing informatics education courses and formulate a set of fundamental recommendations for instructing informatics in accordance with ACPE-accredited pharmacy programmes. | NA | NA | Course Development approach: Evaluation of existing courses, Literature review, and individual experiences  Frameworks used: NA | NA | NA | NA |
| Fuji 2010 (58),  USA,  Conference Abstract | Pharmacy students | Digital literacy course/curricula  The study presented a case analysis of students who participated in an elective course, designed to address the changing demands of pharmacists, and equip them with skills to comprehend and employ nationally accepted health informatics content in practical settings. | In person.  Elective, Standalone course | NA | Course Development approach: NA  Frameworks used: NA | classroom assessment techniques, quizzes, papers, exams, | NA | course evaluation   - Two themes emerged - health informatics is a future career opportunity. - the course is perceived as a unique course of preparation in the students’ program. |
| Frenzel 2010 (59),  USA | Third-year pharmacy students | Electronic Health Records (EHRs)  The Pharmaceutical Care Laboratory III course was enhanced by incorporating disease state management activities using the EHR. This provided an opportunity for students to develop advanced patient-centred care skills. | Blended (using HER)  Core, Integrated | Active-learning, Laboratory activities | Course Development approach: NA  Frameworks used: NA | Completion of SOAP activities | knowledge and attitudes:   - Pre and post survey - significant gains in perceived knowledge and attitude**.** | Opinions:   - Open -ended questions - They reported feeling as if they were working with a real patient and were excited to be able to apply acquired knowledge to care for the patient. - They felt the activities were realistic, practical, and challenged them to think critically. |
| Gardner 2011 (60), USA, Conference Abstract | Students from medical, nurses, pharmacy, allied health, and public health | Digital literacy course/curricula  Throughout the academic year, five seminars were organized, each of which was attended by students from various colleges. These seminars covered topics such as Team-Based Care, the Use of New Technologies in Clinical/Hospital Settings, Ethical and Legal Issues in a World of Electronic Health Records, Patient Safety and Improved Outcomes, and Patient-Centred Care. | Blended (using Technologies in classroom)  Core, integrated into relevant course | Seminars (lecture, and discussion) | Course Development approach: NA  Frameworks used: NA | NA | NA | NA |
| Fox 2011 (61),  USA | PharmD students | Recommendations for educating digital literacy  The authors recommended multiple instructional approaches that could be incorporated into the curriculum to integrate informatics education. They also provided specific examples of pharmacy informatics learning activities, such as e-prescribing, electronic medical records (EMRs), and automated dispensing machines (ADMs). | NA | NA | Course Development approach: consensus-based process  Frameworks used: NA | NA | NA | NA |
| Pantazi 2011 (69), Canada | Students from Health Informatics and Pharmacy | Digital literacy course/curricula  A course was designed to simulate various clinical workflows by providing hands-on experience in the installation and maintenance of a hospital information system (HIS). | Blended,  Core, Standalone | lecture and discussions plus labs and tutorials  Using A Veteran’s Affairs (VA) VistA Hospital Information System (HIS) | Course Development approach: NA  Frameworks used: NA | Practical exam | Performance:   - Analysis of the activity - all students have been able to complete the required tasks. | Opinion:   - Anonymous student feedback - difficulties with VistA textual interface. |
| Kirwin 2013 (62), USA | Third year pharmacy students | Electronic Health Records (EHR)  A simulated hospital pharmacy course was implemented, incorporating patient cases and drug therapy problems that aligned with the students' progression in pharmacotherapy courses. The cases provided a longitudinal perspective for students to identify and recommend solutions within a realistic patient context. | Blended,  Core, Integrated | laboratory simulation,  MEDITECH software package | Course Development approach: NA  Frameworks used: NA | Different students’ activities | Confidence:   - Pre- and post- survey - mean ranks on questions regarding confidence changed   from 1.5 to 2.9 Vs 2.0 to 3.4 indicated increase confidence.  Performance:   - confirmed student achievement of covered competencies. | Opinion:   - Course evaluation survey - few found course content was valuable, - others found it challenging and sometimes difficult to complete in the allotted time. - suggested that more instruction prior to the activities would have been helpful |
| Miranda 2014 (63), USA, Conference Abstract | Third year pharmacy students | Electronic Health Records (EHR)  The aim of the simulation activities was to provide students with an understanding of the components and functionalities of EHRs, and to evaluate their confidence levels in using EMRs before and after the activities. | Blended, | Case-based | Course Development approach: NA  Frameworks used: NA | Completion of SOAP note | Confidence:   - Pre- and post- survey - largest difference found regarding using of paper-based in practice 1.77 vs. 2.26 - confidence in obtaining data from EHR 2.48 vs. 2.93. | NA |
| Leibfried 2014 (64), USA, Conference Abstract | Fist year pharmacy students | Electronic Health Records (EHR)  A simulated rotation module was developed that incorporated an EHR into the institutional pharmacy practice setting | Blended (using web-based material),  Core, Integrated | Case-based  web-based electronic medical record (SimEMR) | Course Development approach: NA  Frameworks used: NA | NA | Experience, Knowledge, confidence:   - Using survey - 83.7% of students felt that SimEMR demonstrated the role of informatics in managing the simulated hospital patients - 78.9% of students enhanced their hospital learning experiences in simulated IPPE. - 72.6%of Students feel more prepared to utilize EMR in upcoming hospital IPPE rotations**.** | NA |
| Cutler 2014 (65), USA, Conference Abstract | All pharmacy students | Digital literacy course/curricula  An online curriculum called "Partners in E" was developed and implemented across multiple pharmacy schools state-wide to introduce pharmacy informatics. | Online,  Either using the modules to develop new standalone electives, or integrating them into existing, required courses and rotations. | NA | Course Development approach: NA  Frameworks used: NA | NA | knowledge, attitudes, and confidence:   - pre-/post-surveys - students showed a statistically significant increase in knowledge, attitudes, and confidence in achieving competencies in pharmacy informatics. |  |
| Manning 2014 (66), USA, Conference Abstract | Second- and third-year pharmacy students | Digital literacy course/curricula  An elective course on health informatics was developed, where students completed exercises focused on various skills related to health informatics, such as data storage, data retrieval, clinical report generation, and networked information system management. | Blended (using technologies in classroom)  Elective, Standalone | NA | Course Development approach: NA  Frameworks used: NA | skill-based exercises | NA | Experience:   - using survey - Quality of course and using technology were rated as high with a mean (standard deviation) response was 4.80 (0.40). - technology was used effectively in the course, mean 4.80 (0.40) |
| Toh 2014 (72),  Singapore, | Final-year undergraduate students | Electronic Health Records (EHRs)  A mobile app was developed that allowed students to access a virtual patient record and engage in one-to-one role-play counselling sessions. The app provided a simulation of real-life patient encounters, with a facilitator guiding the student in solving these cases. | Blended,  Elective, pilot | Case-based  A prototype virtual patient record (VPR) mobile app | Course Development approach: NA  Frameworks used: NA | Case Question involved in VPR | NA | Perceptions:   - using survey - 90.3% of students found the app useful as a teaching aid - 96.7% of students reported that the design of the app features was also understandable and self-explanatory |
| Aungst 2014 (67), USA | NA | Recommendations for educating digital literacy  The letter highlighted the importance of incorporating Health into pharmacy curricula. | NA | NA | Course Development approach: NA  Frameworks used: NA | NA | NA | NA |
| Fuji 2015 (68), USA | All pharmacy students | Digital literacy course/curricula  A course on health informatics was developed as an elective and made available to all pharmacy students. | Online  Elective, Standalone | Lectures, discussion | Course Development approach: Literature Review  Frameworks used:  IMIA, American Association of Colleges of Pharmacy’s Technology in Pharmacy Education and Learning Special Interest Group | Quizzes and examinations | Students’ performance:   - using quantitative metrics via discussion questions, quizzes, written papers, and examinations. - qualitative findings were measured through discussion questions, a goal-setting classroom assessment technique, | End-of-course:   - Students report finding value in the course and recognizing how the knowledge gained could impact their future practice as pharmacists. |
| Rodis 2016 (16), USA | first-year pharmacy student | mHealth  A learning experience for pharmacy students that focused on finding, evaluating, and using medical apps. | Blended (using online material),  Integrated | Pre-recorded lectures, small group workshops | Course Development approach: NA  Frameworks used: NA | NA | Perceptions:   - Pre-post- surveys - perceived improvement in student skills related to: - finding (44% vs 95%), - evaluating (15% vs 93%), - using medical apps in patient care (26% vs 90%) - the health sciences classroom (32% vs 87% after). | Satisfaction of the learning experience:   - using survey - 74% of Students described satisfaction with the educational experience and agreed that it should be repeated in subsequent years |
| Breeden 2016 (17), USA | All pharmacy students | Digital literacy course/curricula  A comprehensive health informatics curriculum was designed with multiple tiers to ensure that all pharmacy graduates acquire essential competencies in this field. The curriculum also offers specialized and advanced training options for pharmacy students. | Blended | Classroom & Experiential education | Course Development approach: NA  Frameworks used: NA | NA | NA | NA |
| Estes 2016 (18), USA | Volunteers’ students from 2nd year Pharmacy and Advanced Practice Registered Nursing students | Tele-pharmacy  A simulation design for conducting telehealth visits was developed, where a student from each program was paired to form an interprofessional (IP) team. The team's objective was to conduct a telehealth patient visit with a standardised patient (SP), incorporating videoconferencing, telehealth monitoring tools, and a simulated academic electronic health record (EHR). | Blended (in person using online technology),  Elective, Pilot | Simulation training | Course Development approach: NA  Frameworks used: NA | NA | Evaluation students’ experience and beliefs:  Qualitive data revealed 3 themes:   - Telehealth Technology is Valuable - Telehealth Technology May Provide Efficient Care - Telehealth Technology Promotes IP Collaboration | NA |
| Hincapie 2016 (19), USA | Second- year PharmD students | Digital literacy course/curricula  A pharmacy informatics program was incorporated into an existence course in drug information (DI) and literature evaluation. | Blended (in person using online technology),  Core, Integrated | Active-learning pedagogy, TBL  Using Partners in E (PinE) | Course Development approach: NA  Frameworks used: NA | TBL will be evaluated on basic concepts learned outside class based on pre-assigned material using a short examination of individual readiness assurance test (iRAT), and (team readiness assurance test, tRAT). | knowledge and attitude:   - Pre- and post-postsurvey - indicated a significant knowledge gain with the greatest absolute percent change in knowledge was related to   - computerized provider order entry (62%, p,0.001)   - electronic health records (41%, p,0.001). - students strongly agreed (19% vs 11%) or agreed (55% vs 31%) to feeling confident in defining basic concepts on health informatics. | NA |
| Leibfried 2016 (20), USA | first year pharmacy students | Electronic Health Records (EHRs)  A simulated EHRs was integrated into institutional pharmacy practice modules as part of the simulated Introductory Pharmacy Practice Experience (IPPE). | Blended (in person using online technology),  Core, Integrated | Case-based approach  Using SimEMRs tool | Course Development approach: NA  Frameworks used: NA | EHR activity (a patient-centred care plan, including assessment, plan, and recommendations for monitoring and follow up in the form of a written SOAP) | Experience, Knowledge, confidence:   - Using survey - 83.7% of students felt that SimEMR demonstrated the role of informatics in managing the simulated hospital patients - 78.9% of students enhanced their hospital learning experiences in simulated IPPE. - 72.6%of Students feel more prepared to utilize EMR in upcoming hospital IPPE rotations**.** |  |
| Miranda 2016 (21), USA | second year pharmacy students | mHealth  The course covered the latest mHealth technologies and devices, and their implications for patient care. | Blended (using technologies in classroom)  Integrated | Workshops consist of lectures, hands-on, and discussions. | Course Development approach: NA  Frameworks used: NA | Completion of worksheet regarding M-health apps. | Perceptions:   - Pre-post survey - Students’ familiarity with mHealth devices increased, 47% to 82% (p<0.001). - No significant change in students’ perception of usefulness of these devices or easily incorporation into practice | NA |
| Nemec 2016 (22), USA | NA | Recommendations for educating digital literacy  The article discusses three significant e-learning resources, namely the Healthcare Information Management Systems Society (HIMSS) Partners in E program, ASHP’s Pharmacy Informatics Essentials program, and the University of Minnesota’s Interprofessional Healthcare Informatics (IHI) program. | NA | NA | NA | NA | NA | NA |
| Fox 2017 (23), USA | Pharmacy students | Recommendations for educating digital literacy  This paper discusses an approach to integrate basic informatics education into pharmacy curricula for all students. | NA | NA | NA | NA | NA | NA |
| Flynn 2017 (24), USA | Pharmacy students | Recommendations for educating digital literacy  An approach to incorporate teaching advanced pharmacy informatics concepts and skills. | NA | NA | NA | NA | NA | NA |
| Fox 2017  (25), USA, | pharmacy and computer science and software engineering (CSSE) students | mHealth  An interdisciplinary approach was taken to develop a course sequence on mobile health (mHealth) and a mHealth application, emphasizing the use of mobile device technology to promote health and wellness. The course involved collaborative efforts across teams to develop a prototype for the mHealth app. | Blended (using technologies (mHealth app) in classroom)  Standalone, one-time course | Lecture and discussions | Course Development approach: NA  Frameworks used: NA | Participation rubric, in-class presentation, peer evaluation rubric | interdisciplinary experiences:   - survey data collected after students entered the workforce - students felt better prepared to work with individuals from other disciplines, (mean 4.82; 5=strongly agree). - using the knowledge from course proposal development in their careers (mean 4.27; 5=strongly agree) | End-of-course:   - End of course - They indicated an overall favourable rating, mean 3.27–3.55; 4=strongly agree |
| Steckler 2017 (26), USA | Pharmacy students | Recommendations for educating digital literacy  The paper focused on the implementation of Health Informatics (HI) in pharmacy curricula and provided recommendations on how and why to do so. | NA | NA | NA | NA | NA | NA |
| Rocchi 2018 (27), Canada , Conference Abstract | NA | Digital literacy course/curricula  An updated version of the "Informatics for Pharmacy Students" e-Resource was released with additional learning activities, building on the previous versions. | Online | NA | Course Development approach: update a previously existence resources  Frameworks used: NA | NA | NA | User testing   - faculty case reports on integration and curriculum changes, learning management system analytics, and feedback from student users - little or no technical difficulties and satisfaction with content and platform. |
| Perkins 2018 (28), USA, Conference Abstract | First Year Pharmacy students | Electronic Health Records (EHRs)  EHRs were integrated into multiple courses to teach students how to identify pertinent patient information and develop sound clinical recommendations over time. | Blended.  Integrated into curriculum | Case- Based | Course Development approach: NA  Frameworks used: NA | Students had to solve 3 series of progressive case scenarios. | Student's performance   - Analysis of the activity - 100% pass rate was obtained | NA |
| Logan 2018 (29), USA, Conference Abstract | second year pharmacy students | Digital literacy course/curricula  A Health Information Technology Skills Lab was developed to emphasize the importance of health technology and provide hands-on training on current and emerging health system informatics. | Blended (using technologies in classroom)  Core, Integrated | discussion, small group hands-on product demonstrations. | Course Development approach: NA  Frameworks used: NA | NA | Knowledge:   - -pre-and post-surveys - statistically significant improvement in understanding of different aspects of health information technology (use, safety, efficiency, cost impact, working knowledge of technology, and benefits to the pharmacy profession). - 97% of students improved their understanding - 89.5% confidence in using health technology | NA |
| Wasynczuk 2018 (30), USA, Conference Abstract | second year pharmacy students | Electronic Health Records (EHRss)  Incorporation of EHR to drug information course that used for completing assignments using EHR | Blended  Integrated | Didactic | Course Development approach: NA  Frameworks used: NA | Assignments | Perceptions:   - Prep and post- survey - a significant increase in students’ knowledge of EHR, (61.4% to 89.2%) - increased in students’ confidence in use of EMR systems, (20.5% to 82.8%). | NA |
| Coons 2018 (31), USA, | second year pharmacy students | Electronic Health Records (EHRs)  A study was conducted where a group of students were given access to a virtual Electronic Health Record (EHR) 48 hours before a scheduled patient encounter, while the control group did not have access to the EHR and were instead given the same baseline information. | Blended (group have online access and group not)  Elective | lectures which precede teamwork activities at the simulator  virtual EHR (DocuCare®) | Course Development approach: NA  Frameworks used: NA | NA | learning efficiency:   - Use of the virtual EHR decreased the amount of time needed to provide the optimal treatment recommendations by 25% compared to control.   Perceptions:   - Surveys showed significantly improved students’ perceptions of their clinical skills, communication, and learning satisfaction. | NA |
| Smith 2018 (33), USA, | Third year pharmacy students | Electronic Health Records (EHRs)  A simulated EHR was implemented to train pharmacy students in the Pharmacists' Patient Care Process and prepare them for using an EHR in clinical practice. The training specifically focused on principles such as collection, assessment, and development of a patient-specific plan. | Blended;  Core, Integrated | Practice Lab and Case Studies  NiaRx® system | Course Development approach: NA  Frameworks used: NA | Completion of SOAP note | Performance:  Comparing grade before and after EHR implementation  did not show a difference in overall student performance on acute patient care and ambulatory care APPE  Perceptions:  Pre-and post- survey showed a significant improvement in students’ perceptions of preparedness to use an EHR in clinical practice when measured by a pre- and post-intervention survey |  |
| VanLangen 2018 (34), USA, | Pharmacy schools | Electronic Health Records (EHRs)  A survey was conducted to investigate the current use of EHRs in the didactic pharmacy curriculum, the perceived benefits of using EHRs to prepare students for pharmacy practice experiences, and future plans for implementing or improving EHR use. | NA | NA | Course Development approach: NA  Frameworks used: NA | NA | NA | NA |
| Skelley 2018 (35), USA | Third year pharmacy students | Electronic Health Records (EHRs)  An elective exercise was developed that incorporated the use of EHRs. The focus was on collecting and assessing patient information from the EHR, developing a plan to optimize pharmacotherapy and achieve goals of therapy, implementing that plan through documentation within the medical record, and identifying the need for monitoring and follow-up. | Blended;  integrated, Elective course | Case-based  Using the cloud-based electronic health record platform, Practice Fusion. | Course Development approach: NA  Frameworks used: NA | Completion of SOAP note | Perceptions:   - Pre- and post- survey - statistically significant changes on three of the questions that dealt with pharmacist skills and knowledge, benefits of an EMR in didactic coursework, and confidence in documenting clinical activities. | Course feedback   - course evaluations and course reflections completed at the end of the semester, - No negative feedback was received on the EMR activity - suggestion indicating additional course time on interpreting and applying practice guidelines to individual patients and patient cases would be beneficial. |
| Darley 2019 (32), USA | Pharmacy students | Digital literacy course/curricula  A 4-semester course series was developed with the aim of enhancing pharmacy students' knowledge of informatics. The course covered topics such as the role of technology in the medication use process, pharmacy operations, quality and safety of care, and cost reduction. | Blended (using technologies in classroom)  Core, Standalone | Skills laboratory | Course Development approach: Pilot Course  Frameworks used: NA | NA | Perceptions:  (Result from class survey in 2017 and 2018)   - Pre- and post- survey s - majority of students strongly agreed or agreed that interaction with the vendors improved their understanding of automation/informatics products (97% in both 2017 and 2018) - their confidence in using automation/informatics products also improved (89% in 2017 and 86% in 2018) in the hospital environment | NA |
| Gibson 2019 (36), USA | Second year pharmacy students | Electronic Health Records (EHRs)  A simulated EMR was created for 2nd year pharmacy students in a case-based application course. | Blended;  Core, integrated | Case-based  Pilot simulated EMR (SEMR) Microsoft PowerPoint | Course Development approach: NA  Frameworks used: NA | Completion of SOAP note | Perceptions:   - Pre-survey and post-survey in two consecutive courses between 2016 and 2017. - indicated that perceived confidence and efficiency navigating EMRs improved after the activity - no significant differences regarding students' perception of the benefits | NA |
| Martin 2019 (37),USA | AACP members (American Association of Colleges of Pharmacy) | Recommendations for educating digital literacy  The AACP task force developed a list of pharmacy informatics competencies by consulting 11 sources. Their recommended informatics course content includes basic computer competency with a focus on web technologies such as protocols, security, and socio-political aspects like privacy, theoretical understanding of information technology, and applications such as digital communication methods like email and productivity tools like word processors, spreadsheets, presentations, and calendars. | NA | NA | Course Development approach: literature searches, existence courses, and expert consultation via tow focus group  Frameworks used: Technology Informatics Guiding Education Reform (TIGER) | NA | NA | NA |
| Pontefract 2019 (38), UK | Academics & representatives from NHS England, NHS Digital & EPR system providers | Recommendations for educating digital literacy  A national working group of academics in the UK was established to develop a list of competencies and learning outcomes. Six domains were proposed - working as a practitioner in the digital healthcare environment, accessing data, communication, generating data, multidisciplinary working, and monitoring and audit. | NA | NA | Course Development: expert consultation via eDelphi  Frameworks used: NA | NA | NA | NA |
| Carr-Lopez 2020 (39), USA | First- and second-year pharmacy | Electronic Health Records (EHRs)  A medication safety module was developed and integrated into an Introductory Pharmacy Practice Experience (IPPE) program. The module focused on medication safety and involved reviewing electronic health record (EHR) patient records and entering required laboratory tests. | Blended (using online technology),  Core, Standalone | experiential training  remote access to EHRs VA Northern California Health Care System (VANCHCS) | Course Development approach: NA  Frameworks used: NA | NA | Perceptions:  Pre- and post- survey showed improvement in:   - Explain the role of a clinical dashboard to conduct population health (Strongly Agree/Agree 7 Vs 47) - use an actual (non-simulated) EHR to perform patient care (Strongly Agree/Agree 12 Vs 53) - ability to monitor laboratory tests, (Strongly Agree/Agree 10 Vs 54) | NA |
| Ives 2020 (40), USA | Second year pharmacy students | Electronic Health Records (EHRs)  An EHR simulation was implemented in a health system-based skills laboratory course. Students were able to practice medication order verification during two laboratory sessions. | Blended;  Core, integrated | practice laboratory sessions  Platform EHR Go! | Course Development approach: NA  Frameworks used: NA | practical examination, | Performance:   - Analysis of activity - improved significantly compared with that of those who completed the activity the previous year using a paper-based medication form.   Confidence and perceptions:   - The confidence levels of students who used the EHR doubled and in some cases tripled pre- and post-EHR implementation. | NA |
| O'Connor 2020 (41), USA | Pharmacy students | Tele-pharmacy  A course called "Exploration of Telemedicine" was designed to train pharmacy students by involving incarcerated patients who are receiving HIV/Hepatitis C care through telemedicine. The course aimed to provide students with the knowledge and skills to better understand and utilize telehealth in their future practice. | Blended  Elective, standalone | online modules, discussion and observe telehealth clinical activities in real time. | Course Development approach: NA  Frameworks used: NA | NA | NA | NA |
| Vlashyn 2020 (42), USA | first, second-, and third-year pharmacy students | Electronic Health Records (EHRs)  The study aimed to integrate EHRs into pharmacy curricula with the primary objective of providing students exposure to an EMR during their didactic training before pharmacy practice. The study also focused on gathering students’ perspectives regarding the integration of EHRs. | Blended (using online material);  Integrated into curricula | NA | Course Development approach: NA  Frameworks used: NA | NA | Perceptions:   - using survey and Qualitative data (focus group) - 48.7% of survey respondents altogether agreed or strongly agreed that using the EHR enhanced their learning in pharmacy classes and laboratories. - Qualitative data were organized into four themes:   - current priorities for use within the pharmacy curriculum  - EHR benefits  - EHR barriers  - future priorities for EHR use to prepare students for pharmacy practice. | NA |
| VanLangen 2020 (43), USA | Pharmacy schools in USA | Electronic Health Records (EHRs)  The survey focused on the general use of EHRs in schools and colleges of pharmacy (S/COP) and characterized EHRs as a tool to assess students' patient care skills in the didactic curriculum. | NA | NA | NA | NA | NA | NA |
| cook 2021a (45), USA | Pharmacy students at University of Nebraska Medical Centre (UNMC) and at the University of California San Diego | Electronic Health Records (EHRs)  This study involved the participation of two schools of pharmacy. Electronic health records (EHRs) were implemented to teach EHR skills such as documentation, medication order evaluation, and development of patient therapeutics plan. | Blended (Using online EHR)  Integrated | EPIC Education | Course Development approach: NA  Frameworks used: NA | NA | self-reported comfort:   - using survey from two schools - students were more comfortable in finding information compared to entering new information in the EHR. - Students were most comfortable reviewing laboratory information, progress notes, and medication lists and less comfortable performing medication reconciliation and identifying clinical errors. | NA |
| Cook 2021b (44), USA |  | Recommendations for educating digital literacy  The study examined the utilization of electronic health records (EHRs) in pharmacy It provides an overview of EHR activities that were incorporated into courses and highlights examples of the competencies and skills that were taught and assessed using EHRs | NA | NA | NA | NA | NA | NA |
| Mantel-Teeuwisse 2021 (46) | academics, pharmacy schools, pharmacists and pharmacy students | Recommendations for educating digital literacy  The International Pharmaceutical Federation (FIP) undertook a worldwide survey on the integration of digital health into pharmacy education, using an online questionnaire. | NA | NA | NA | NA | NA | NA |
| Neumann-Podczaska 2021 (47) | Medical and Pharmacy Students all in the last year of their curriculums. | Tele-pharmacy  The project aimed to offer practical learning opportunities in tele-pharmacy to medical and pharmacy students. The emphasis was on interprofessional medication management, with students working collaboratively under supervision to provide care to vulnerable older individuals (aged 60 years and above) who had limited access to healthcare services. | Blended - students and supervisors in person together, remotely contacting patients. | Consultations  Interned-Telephone Consultation Services (ITCS) | Course Development approach: NA  Frameworks used: NA | NA | perceptions:   - In-depth interviews - Students reported improvement in their self-confidence and understanding of patient care skills, Ability to conduct a literature review, communication and telehealth skills, and the interprofessional collaboration skills | NA |
| Perkins 2021 (48),USA | 1st, 2nd and third year pharmacy students | Electronic Health Records (EHRs)  curriculum-wide implementation of a simulated EHR software  A simulated EHR software was implemented throughout the pharmacy curriculum, with a focus on four competencies: two related to patient counselling in the first year, and two related to order verification and medication error reduction in later years. | Blended (in person using online technology),  integrated | Case-based  EHR Go | Course Development approach: pilot elective course  Frameworks used: NA | NA | No student data were collected | NA |
| Reynolds 2021 (49),USA | Pharm.D. Candidates in a clinical capstone course. | Electronic Health Records (EHRs)  An EHR was introduced in an online capstone course to help students gain familiarity with the pharmacist's patient care process (PPCP) and using an EHR in clinical practice. | Online  Integrated | NA | Course Development approach: NA  Frameworks used: NA | .NA | Perception:   - Using survey - Students agreed the EH R conferred value in data collection processes (4) (median Likert score) assessing therapy and disease (4) plan composure (5) and monitoring/follow-up (5) - Student-perceived value of the EHR was high for both inpatient (5) and ambulatory care (4) cases and students felt better-prepared for experiential rotations with an EHR (4).   Performance:   - There were no significant differences in academic performance before and after EHR implementation. | NA |
| Wasynczuk and sheehan 2021 (50), USA | Pharmacy students at the Purdue University College of Pharmacy | Electronic Health Records (EHRs)  Incorporating a teaching Electronic Medical Record (EMR) into a Drug Information (DI) assignment, students were asked to use the EMR to submit a patient-specific written response addressing a drug-related question.  . | Blended (using online technology);  Core, integrated | lecture and active-learning discussion  a web-based platform that includes medical records of real patients from historic hospital records. | Course Development approach: NA  Frameworks used: NA | Assignments | Perceptions:   - pre-post survey - Differences were found in respondents who agreed that didactic use of an EMR increased their confidence in obtaining information from an EMR (20.5% to 82.8%) - improved their knowledge of EMR systems (61.4% to 89.3%). | NA |
| Ngo 2022  (51), USA | USA pharmacy schools | Digital literacy course/curricula  The study aimed to identify and describe PharmD/MSHI dual degree programmes offered at Schools and Colleges of Pharmacy (S/COP) in the United States. This was achieved by conducting a thorough search of various sources, including Pharmacy School Admission Requirements websites, the Pharmacy College Application Service website, and individual websites of S/COPs. | NA | NA | Course Development approach: NA  Frameworks used: NA | NA | NA | NA |

**Supplemental Table 5. Educational interventions, skills covered, and outcomes of each study included in the review**

| **Study ID (first author and year of publication),** | **Intervention- a brief description of the educational intervention** | **Aim of the educational intervention** | **Intervention structure- Material used, educational strategies (e.g., tutorials, lectures, online modules), modes of delivery (e.g., face-to-face, internet or independent study package), instructor(s) involved** | **Skills Covered the educational intervention** | **Assessment used** | | **Outcomes** |
| --- | --- | --- | --- | --- | --- | --- | --- |
| Mihalas 1997(70), | Introducing a pharmaco-informatics course to pharmacy students. | Familiarised with basic concepts, terminology, and application of informatics in practice  Aware of role of pharmacist in informatics.  Acquire basic skills in using computers, for documentation, data bases and some specific applications in pharmacy. | Material:  Computers, software devoted to specific topics e.g., drug design, pharmacokinetics, and pharmacodynamics.  Educational strategies: Lectures and Lab works  Delivery:  Face-to-face  Environment:  Classroom settings, University of Medicine, and Pharmacy  Schedule: 15 hours of lectures and 15 hours of laboratory works, | Familiarised with fundamental health informatics concepts.  Technical Proficiency in using technologies for documentation, data bases and some specific applications in pharmacy. | No assessment details were reported. | | NA |
| Ab Rahman 2002(71), | Introducing a new course for pharmacy students called “Pharmacoinformatic,” which combines drug information and pharmacy information systems. | Improve students’ awareness concerning the impact of information technologies on pharmacy practice and drug usage.  Preventing the occurrence of "computer phobia" in pharmacists. | Material: reading materials, computers.  Educational strategies: Lectures  Modes of delivery:  face-to-face  Instructors:  Clinical pharmacy lecturers (x 4-5) per session  Environment: Classroom and clinical settings, School of Pharmaceutical Sciences in Malaysia and training at a drug information centre situated at a nearby general hospital  Schedule: two-unit course | Using digital technology and tools for personal learning, such as computerised drug databases; and evaluating literature.  Familiarised with role of computer-assisted information management in the health care system. | **Group project** (n=15 to18 student) using computers, included the evaluation of pharmacy related Internet sites and the establishment of group Web sites | 40% on coursework | Total 173 students surveyed using Questionnaires  Knowledge about pharmacoinformatic increased from 26.6% before to 77.3% of after the course.  students were satisfied with the course and found it up to date 64.6%, should be maintained 79.9%.  students were also satisfied with coursework appropriateness 72.3%  70.8% reported facilities insufficient e.g., Computers. |
|  |  |  |  |  | Individual assessment | 60% on essay final examinations |  |
| Seifert 2002(52), | A tele pharmacy teaching and service model that serves as a training tool for pharmacy students and as a mechanism for the delivery of pharmaceutical care in the rural community | providing pharmacy students educational opportunities that will better prepare them for a unique practice environment. | Material: network system, pharmacy computer system, 2way video and audio transmission capabilities.  Educational strategies:  Lectures and a “hands on” experience.  Modes of delivery:  Face to face and online  Instructors:  Faculty from the Centre for Telemedicine (x1) per lecture  Physician operating the telemedicine (x1) per ‘hands on’ experience  Emergency medical technician delivering health care services in rural area (x1)  Pharmacist provided tele pharmacy (x1)  Environment:  Classroom and clinical settings, school of pharmacy at Lubbock, Physician’s office in Plainview, Tex, remote sites in Turkey, Tex, and Quitaque, Tex,  Schedule:  3^rd^ year: 6-hour didactic session  4^th^ year: 1-week rural clerkship for | Using digital technologies to communicate with patients and other people.  Technical proficiency in providing tele-pharmacy service. | **Individual assessment**: daily documentation of the activities on a log sheet. | Assessment of documentation’s quality | Total of 14 student completed the experience.  Assessment of **Documentations** quality, most of their activities were poorly documented  Evaluation of the course using survey, found that mean evaluation of didactic courses was excellent (2.82/3.0), and clerkship evaluations were fair to good (2.64–3.62/5.0) |
|  |  |  |  |  | students’ performance on tele- pharmacy | Live evaluations |  |
| Brown 2005 (53), | An Internet-based medical chart (IMC) developed for documentation of simulated patient care follow-up activities that used live actors as patients. | Introduce IMC as an integrated documentation and evaluation tool that will accommodate, multiple learning activity and promote efficient feedback | Material: an IMC system required 4 components: a database, dynamic Web-page skeletons, a server, and computers with Internet access.  Educational strategies:  Blended.  Modes of delivery:  Face to face  Instructors:  Faculty members  Environment:  Classroom setting  Schedule: 13 weeks course, 5 weeks focused on developing clinical patient-centred care skills. | Using EHRs to find, manage, organise, store, and share patient data.  Proficiency in using EHRs for documentation. | Group activity:  5 "Finish the SOAP note"  students worked in pairs to review previous SOAP (Subjective/Objective, Assessment, and Plan) note documentation from simulated provider(s) in the IMC system, complete a follow-up interview and assessment of the simulated patient, develop the care plan, deliver the care plan to the patient including providing necessary education, and document the simulated patient care activities in a SOAP note in the IMC system. | Using the rubric in the IMC system, an instructor evaluated the components of background information, subjective and objective data, assessment, and plan  assigning the ratings of exceptional (EX), satisfactory (SA), or needs improvement (NI) as dictated by the rubric | Total of 98 students' performance were analysed, identified challenging areas, including incomplete documentation of therapeutic goals and specific follow-up laboratory test recommendations.  95.5% of students prefer using EHR compared to paper- based system,  91% of the students reported ease of use. |
| Zagar 2007 (55) | Implementation of a teaching module that prepares students to assist Medicare beneficiaries in evaluating and enrolling in Medicare Part D plans. | provide students with an understanding of the basic programme structure.  assist students in selecting a drug plan by guiding them through the options offered. | Material: web-based Medicare beneficiaries, PowerPoint presentation and reading material “Medicare & You.”  Educational strategies: Lecture and Active learning  Modes of delivery: face to face  Instructors:  Educator (x1) per lecture  Environment: Classroom settings.  Schedule: 6-hours module on 3 sessions.  First: Lecture about low-income provisions, formulary requirements, and enrollment information for Medicare Part D.  Second: facilitated online navigation through the Medicare Prescription Drug Plan Finder.  Last: active learning included assessments activity. | Exposure to digital technology e.g., Medicare Part D.  Proficiencies in using Medicare Part D to evaluating and enrolling patients. | Groups activity:  develop a list of ‘‘The Top 10 Things Every Medicare Beneficiary Should Know About Medicare Part D.’’ and 10-minutes mock counselling sessions, | assessed by students’ peers. | Total of 64 student completed the course  Analysis students’ performance, average scores on the 3 activity was above 90%.  Students’ responses using survey were uniformly positive, with the scores ranging from 4.6 to 4.8., |
|  |  |  |  |  | Individual activity:  Select an appropriate Medicare drug plan for the given drugs and write a paper comparing the 3 plans on price comparisons and justifications | assessed appropriateness by the instructors |  |
| Brown 2007 (54), | integrate an Internet-based medical chart (IMC) system into a pharmacotherapy course to provide students with the subjective and objective components of a note and asks them to complete the assessment and plan for a simulated patient case. | offered students with the opportunity to strengthen their documentation skills  Identify the difficulties that students frequently encountered when attempting to improve their documentation and clinical thinking skills. | Material: the IMC system.  Educational strategies:  Blended.  Modes of delivery:  Face to face and online using IMC.  Instructors:  Evaluators (x9)  Environment:  Classroom settings.  Schedule: integrated into a course with 4 modules. | Using EHRs to find, manage, organise, store, and share patient data.  Proficiency in using EHRs for documentation. | Individual activity:  Four ‘‘finish the SOAP note’’ activities each completed within a week. | Assessment using rubric  100% an excellent (EX) rating,  85% for a satisfactory (SA) rating,  70% for a needs improvement (NI) rating.  0% markedly lacking, little or no effort into the exercise. | 124 students surveyed, 86.3% reported improved their pharmacotherapy knowledge, 78.2% documentation skills  87% of the students avoided repeating previous mistakes by their final documentation activity.  Evaluator’s opinions were assessed using survey n=9, Overall agreements that system easy to use  felt the exercises fostered improvement in both pharmacotherapy knowledge and documentation skills |
| Cutler 2008 (56),  USA, | Introducing a Medicare Part D finder tool to the course of “Health Policy for Pharmacists course.” to introduce the organization, financing, and delivery of health care in the United States. | Improving students' understanding of Medicare Part D.  enhance student' skill and confidence in using the online Medicare Prescription Drug Plan Finding tool. | Material: online Medicare Plan Finder tool.  Educational strategies:  Blended (lectures, exercises)  Modes of delivery:  Face to face  Instructors:  Faculty member (x1)  Clinicians (x2), evaluate the appropriateness of Part D plans for Medicare beneficiaries in clinical settings.  Teaching assistants (x3) per lecture.  Environment:  Classroom setting, University of California, San Francisco (UCSF) School of Pharmacy  Schedule:  Two lectures for 90-minute on benefit and effective use of the Medicare Part D. | Exposure to digital technology e.g., Medicare Part D.  Proficiencies in using Medicare Part D to evaluating and enrolling patients. | **Individual assessment**: Identify correctly the 3 least expensive plans and corresponding costs. | Assessed based on the accuracy of the plan and/or the cost before and after the course. | 114 students who completed both the pre- and post-test.  All general knowledge questions about Medicare Part D showed significant improvement; however, the usage of the Medicare Plan Finder question showed the largest improvement, with correct answers increasing from 23.7% to 83.3%.  a significant increase in Mean (SD) of student confidence in using Medicare Part D from 2.9(1.3) to 4.3 (0.6)  Students' measured attitudes did not alter in general, except for the role of Pharmacists as patient advocates, which increased from 3.8 (0.9) to 4.5 (0.7). |
| Frenzel 2010 (59),  USA | Adding an EHRs activity to the pharmaceutical care laboratory course, which included disease state management activities in which each student was assigned a unique patient case, with the complexity of each case increasing throughout the semester. | Using EHRs in disease state management activities to aid pharmacy students in honing their skills in patient-centered care. | Material: Electronic Medical Records, reading material (national treatment guidelines or review articles.)  Educational strategies:  Blended  Modes of delivery:  Face to face  Instructors:  pharmacist faculty (x4) per lecture  Environment:  Classroom settings, The North Dakota State University  Schedule:  1 hour/week lecture and didactic coursework. | Using EHRs to find, manage, organise, store, and share patient data.  Proficiency in using EHRs for documentation. | Individual activity:  longitudinally followed one simulated case: Review patient medication, design and monitor evidence- based patient centred care plan and document SOAP note in EHR system. | Verbal feedback during laboratory  Written feedback via a SOAP note grading rubric which evaluated students written patient assessment, goals of therapy, medication and monitoring recommendations, and follow up. | Pre-course and post-course surveys were completed by 89 and 66 students, respectively.  Significant improvements in perceived knowledge were found by asking questions about:  pharmacists had the skills and resources to help patients with their drug therapy problems. (p= 0.027)  having access to a patient's EHR would be helpful for documenting patient care activities(p=0.009)  having access to a patient's EHRs would enable pharmacists to play a larger part in the healthcare team. (p=0.003) |
| Pantazi 2011 (69), Canada | A health informatics course simulated various clinical workflow by installation and maintenance of a hospital information system (HIS) into audience from Health informatics and pharmacy.  This included Setting up virtual printing and allowing health care professionals to role play for clinical workflow simulation. | Improve student learning and experimentation with current clinical information systems. | Material: Veteran’s Affairs (VA) VistA Hospital Information System (HIS),  Educational strategies:  Blended  Modes of delivery:  Face to face  Instructors:  pharmacist faculty (x4)  Environment:  Classroom settings, Conestoga College, Health Informatics University of Waterloo, Pharmacy  Schedule:  2 hours/week lecturing and discussions  2 hours/week labs and tutorials | Familiarised students with digital technology e.g., hospital information system | Individual practical exam:  1. using their individual VA pharmacist account, adding, accepting, and verifying new inpatient medications ordered for an existing patient of their choice.  2. managing the inpatient pharmacy by printing and analysing administrative reports. | Task completion was demonstrated by medication profiles and activity logs to be printed and submitted using an electronic drop-box. | All students have been able to complete the required tasks  Anonymous student feedback, reported difficulties with VistA textual interface. |
| Kirwin 2013 (62), USA | A course on simulated hospital pharmacy was introduce using simulated patient cases and Drug-therapy problems  . | Develop a simulated hospital pharmacy module.  Enhance students' confidence and skills to conduct tasks a pharmacist might encounter. | Material: The MEDITECH software EHR.  Educational strategies:  Blended  Modes of delivery:  Face to face  Instructors:  faculty members (x 2)  pharmacists (x3)  Environment:  Classroom settings, Northeastern University.  Schedule:  The module taught over 5 weeks. | Using EHRs to find, manage, organise, store, and share patient data. | **Individual assessment:** longitudinally followed two simulated cases: complete a medication reconciliation worksheet; compile relevant patient information in a pharmacist’s monitoring form; identify any drug therapy problems and provide recommendations for resolution and completed a discharge worksheet using a paper form | Assessment based on correctness using an established key. | 110 students completed pre- and post- survey  Increase in student confidence in using EHRs, mean ranked changed from 1.5-2.9 (low comfort/confidence) to 2.0-3.4 (moderate comfort/confidence). |
| Miranda 2014 (63), USA, Conference Abstract | Integrating Electronic Medical Records (EMR) into a College of Pharmacy Curriculum, | familiarize students with components and functionalities of EMRs | Material:  EHRs  Educational strategies:  Blended  Modes of delivery:  Face to face  Environment:  Classroom settings | Using EHRs to find, manage, organise, store, and share patient data.  Proficiency in using EHRs for documentation. | **Induvial assessment:** completion of SBAR notes (situation, background, assessment, and recommendation) for eight simulations. | Evaluating confidence pre and post completion of 8 cases. | 50 students completed the pre-survey, and 27 students completed the post-survey,  largest difference found regarding preferences in using paper-based report to EHRs in practice (1.77 vs. 2.26)  confidence in presenting patients’ data from EHR (2.48 vs. 2.93). |
| Leibfried 2014 (64), USA, Conference Abstract | Incorporated a EHR into the institutional pharmacy practice setting modules within the simulated rotation. | Evaluate the value of a commercially accessible simulated EHRs in a simulated rotation. | Material: web-based electronic medical record (SimEMR)  Educational strategies:  Blended  Modes of delivery:  Face to face  Environment:  Classroom settings, St. John’s University, | Using EHRs to find, manage, organise, store, and share patient data.  Proficiency in using EHRs for documentation | No assessment details were reported. | | Survey on experiences and confidence in using EHR completed by 270 students.  83.7% of students felt that SimEMR demonstrated the role of informatics in managing the simulated hospital patients.  78.9% of students felt that using EHRs enhanced their hospital learning experiences.  72.6% of students feel more prepared to utilize EHR in upcoming hospital IPPE rotations. |
| Manning 2014 (66), USA, Conference Abstract | Development of an elective course on health informatics, the course outlined the history and development of informatics, the components and functions of medication management systems, the processes of medication ordering systems, and the evolving technologies impacting pharmacy. | Empower students to leverage technology to improve healthcare outcomes. | Educational strategies:  Blended  Modes of delivery:  Face to face  Schedule:  two-credit elective course | Familiarised with fundamental health informatics concepts | Students completed skill-based exercises on the storage of data, retrieval of data, generation of meaningful clinical reports, and management of networked information systems.  Assessment not reported. | | Elective involved 8 students.  Survey completed by 5 students.  Quality of the course and use of technology rated high, with a mean (standard deviation) response of 4.80 (0.40).  Technology was used effectively in the course, with a mean (standard deviation) response of 4.80 (0.40). |
| Toh 2014 (72), | Introducing a virtual patient record mobile app to educate pharmacy students on the types of PHIS available from EHRs. | Develop a mobile app prototype for a virtual patient record (VPR).  Educate students about the data that EHRs can provide. | Material: virtual patient record (VPR) mobile app  Educational strategies: Blended  Modes of delivery:  Face to face  Instructors:  1x faculty per class  Environment:  Classroom settings, Department of Pharmacy, National University of Singapore (NUS),  Schedule: 1 class/ week for 4 weeks. | Using digital technology to find, manage, organise, store, and share patient data. | **Induvial assessment:** Role-play sessions with a facilitator mimicking real-life patient:  Using the VPR to solve the clinical case scenarios available through the “Case Questions” feature. | Assessment was not reported | Survey on the usefulness of the app completed by 31 students.  90.3% of students found the app useful as a teaching aid.  96.7% of students reported that the design of the app features was understandable and self-explanatory. |
| Fuji 2015 (68) | An elective course on HI available for all pharmacy students.  The course was consisted of weekly Voiced-over PowerPoint lectures and short discussion questions were embedded in each lecture. | Address the growing use and utilisation of health technology, as well as the insufficient coverage of this content. | Material: voiced-over PowerPoint lectures, required readings each week  Educational strategies: Lecture, discussion  Modes of delivery:  Online  Environment:  Online platform at Creighton University School of Pharmacy and Health Professions  Schedule: 2-credit, semester length course | Familiarised with fundamental health informatics concepts | Assessment was divided on  (10%) discussion questions, reflection on discussion questions.  (10%) quizzes, 10 multiple choices covered key content of lectures.  (20%) papers, 5 paper literature review to allow students explore a research problem impacted by HIT.  final examinations (60%). |  | First 5 course offerings (2009-2013) had 39 students completing the course.  Students scored an average of 92% overall in the course.  No significant differences were observed in average scores across the 5 course offerings. |
| Rodis 2016 (16) | A novel learning experience for pharmacy students that focused on finding, evaluating, and using medical apps.  Students listened to a recorded, Web-based lecture on finding, evaluating, and using mobile apps in patient care. Followed by interactive workshop which students’ discussion on strategies for finding and using apps in health care. | Educating students to use mHealth and evaluating the apps effectively. | Material: pre-recorded, Web-based lecture, tool for evaluating medical apps and list of medical apps.  Educational strategies:  Didactic, workshop, and project-based experiences  Modes of delivery: Online and face to face  Instructors:  Faculty member, (x1) per lecture  Faculty member, (x1) per discussion workshops  Environment:  Online platform and classroom setting  Schedule:  38-minute online lecture  2-hours small group workshop | Exposure to digital technology e.g., mHealth  Proficiency in using digital technology e.g., mHealth | Group activity:  Evaluation of 6 medical apps using the evaluation tool by (4 to 5 students) and discussed with each group. | Survey used to evaluate the effect of these medical apps on learning experience. | Postsurvey completed by 119 students.  Perceived improvement reported in student skills related to finding (44% to 95%), evaluating (15% to 93%), and using medical apps in patient care (26% to 90%) in the health sciences classroom (32% to 87%). |
| Estes 2016 (18) | implement an educational experience that introduced students to telehealth technology in a simulated IP clinical environment. | Familiarised students with telehealth technologies.  Foster interprofessional (IP) education for nursing and pharmacy students | Material: videoconferencing, telehealth monitoring tools, and a simulated academic EHR.  Educational strategies:  Blended  Modes of delivery: Online and face to face  Instructors:  Standardised patient (x1) per simulated activity  Faculty member (x1) per session  Environment:  Three remote locations, the students and Patient accessed a videoconference meeting and conducted a simulated IP telehealth visit. | Using digital technologies to communicate with patients and other healthcare professionals.  Technical proficiencies in digital technology utilization e.g., telehealth, EHR | IP Activity:  An IP Telehealth Visit learning experience, the students and patients accessed a videoconference meeting and conducted a simulated IP telehealth visit to demonstrate use of telehealth monitoring tools (blood pressure, weight scale, SpO2, and pulse monitors) | No assessment reported.  Survey students about their experience | Simulation activity involved 15 students.  Three themes emerged from the qualitative data:  Telehealth technology is valuable.  Telehealth technology may provide efficient care.  Telehealth technology promotes interprofessional collaboration. |
| Hincapie 2016 (19), USA | A pharmacy informatics program incorporated into an existence course in drug information (DI) and literature evaluation using Team-based learning approach. | provide students with a fundamental understanding of pharmacy informatics and Health technology, | Material: online curriculum, known as Partners in E (PinE),  Educational strategies:  Team-based learning  Modes of delivery: Online and face to face  Environment:  Online platform and Classroom setting, California Northstate University College of Pharmacy.  Schedule: 3 hrs /week for five consecutive weeks.  Class time was divided among  15min individual readiness assurance test (iRAT),  15min team readiness assurance test (tRAT)  30 minutes mini-lecture  90 minutes application exercises | Familiarised with basic concepts, terminology, and application of informatics in practice | Induvial assessment: iRAT  Group assessment: tRAT | students are evaluated on basic concepts learned outside class based on preassigned material. | Pre-and post-survey completed by 83 students.  Significant knowledge gain observed, with the largest absolute percent change related to computerized provider order entry (62%, p<0.001) and electronic health records (41%, p<0.001).  Improvement observed in confidence levels for defining basic health informatics concepts, increasing from 31% to 55%. |
| Leibfried 2016 (20), | Introduced EHRs into the simulated Introductory Pharmacy Practice Experience (IPPE) course, where each student access EHR to follow-up and provide patient –centred skills for simulated cases. | Provide activities to simulate both community pharmacy and institutional pharmacy practice settings. | Material:  web-based simulated electronic medical record (SimEMR^®^, KbPort LLC, Allison Park, PA)  Educational strategies:  experiential training, Case-based activity  Modes of delivery: face to face  Instructors:  faculty members (x2) per class  Environment:   St. John’s University College of Pharmacy and Health Sciences.  Schedule: over 13-week | Using digital technology e.g., EHR to find, manage, organise, store, and share patient data. | Individual assessment:  Longitudinal case follow-up, students were asked to submit a written Subjective, Objective, Assessment, and Plan (SOAP note on paper) | received verbal and written feedback | 365 students participated in the activity  83.7% of the students felt that the simulated EMR demonstrated the role of informatics in managing simulated hospital patients  78.9% of the students felt that the simulated EMR enhanced their hospital learning experiences |
| Miranda 2016 (21), | introduction of an mHealth activity into the Medical Informatics and Technology course which focused on appropriate use of mobile technology through evaluation and use, integration of mHealth technology into pharmacy practice and potential professionalism issues associated with their use.  Students worked with wireless blood pressure cuffs, wireless scales and smart body analysis, personal health devices and wireless glucometers | Introduce students to mHealth technology and their role in healthcare | Material:  web-based simulated electronic medical record (SimEMR^®^, KbPort LLC, Allison Park, PA)  Educational strategies:  Lectures and discussion  Modes of delivery: face to face  Instructors:  faculty members x2  pharmacist x1  APPE last year students x2 per workshop.  Environment:   St. John’s University College of Pharmacy and Health Sciences.  Schedule: two sessions that were 2 weeks apart  1^st^: 30 min lecture, 20min group activity per station  2^nd^: 30 summary lectures | Exposure to mHealth technology | Group activity:  students were asked to submit a worksheet which had several questions that guided students through the evaluation of specific mHealth devices. | No details on assessment | 104 students completed preworkshop survey and 96 students completed postworkshop survey  Familiarity with mHealth devices increased from 47% to 82% (p<0.001 |
| Fox 2017  (25), | an interdisciplinary course in mobile health (mHealth) was introduced to pharmacy and computer science and software engineering students.  The focused was to collaborate within and across teams to develop an mHealth app prototype and used a systematic method to develop syllabi for an undergraduate, professional, and graduate level course series focused on mHealth. | Provide a unique opportunity for a collaborative student learning experience.  Allow pharmacy and computer science and software engineering (CSSE) students to use mHealth teaching, research, and outreach as the unifying themes of their interactions. | Educational strategies:  lecture, interactive discussions  Modes of delivery: face to face  Instructors:  faculty members, from CSSE and pharmacy (x2) per course  Environment:   Auburn University  Schedule: 2-hours/week for 17 weeks. | Exposure to mHealth technology  Ability to develop new mHealth app prototype working in collaboration with other.  use of digital technologies e.g., mHealth for personal learning and professional development | **Group activity:** developing and presenting a course proposal, | 30-min “pitch” presentation | 11 pharmacy students and 5 computer science students were enrolled in the course.  Pharmacy students rated the course favourably with a mean score of 3.27-3.55 out of 4.  In a survey after entering the workforce, students felt better prepared to collaborate with individuals from other disciplines (mean 4.82 out of 5) and use the knowledge from the course in their careers (mean 4.27 out of 5). |
|  |  |  |  |  | building a purchase plan for mHealth equipment | Completed syllabus with quality assessment |  |
|  |  |  |  |  | developing an mHealth app prototype, | software app with quality assessment |  |
|  |  |  |  |  | delivering a disease state presentation (pharmacy students only), | In-class presentation |  |
| Coons 2018 (31), | Integration of virtual HER and simulate patients experiences into pharmacy curriculum to assess its efficacy. | Using a virtual EHR and patient simulation to improve clinical skills, communication skills, and students’ satisfaction. | Material: virtual EHR (DocuCare®), patient simulator used was SimMan  Educational strategies: blended teaching (lectures and simulation-based learning using high-technology mannequins)  Mode of delivery: Face to face  Environment: Peter M. Winter Institute for Simulation Education and Research (WISER). | Using EHRs to find, manage, organise, store, and share patient data.  Proficiency in using EHRs for documentation. | Group activity:  Students were randomized to an intervention or control group of three disease state-specific practice patient scenarios.  A group of students had access to the virtual EHR 48hrs before a scheduled patient encounter. The control group did not have access to the EHR and instead were given the same baseline information through PowerPoint slide. | Assessments were graded using an objective pharmacotherapy rubric, evaluated include patient introduction, data collection/interpretation, problem list, pharmacotherapy plan, monitoring, and verbal communication. | 102 students completed pre- and post-surveys.  Use of the virtual EHR reduced the time required to provide optimal treatment recommendations by 25% compared to the control group.  Surveys revealed a significant improvement in students' perceptions of their clinical skills, communication abilities, and satisfaction with their learning experience. |
| Smith 2018 (33), USA, Original research | A simulated EHR was introduced to third-year pharmacy students in a practice lab and case studies course series. The impact of the simulated EHR was measured by comparing student grades from acute patient care and ambulatory care advanced pharmacy practice experiences (APPEs) before and after EHR implementation. | prepare students to use an EHR in clinical practice. | Material: EHRs (NiaRx®), Microsoft Excel (mock EHR)  Instructors:  multiple instructors with a ratio of 1:6  Educational strategies:  Blended (practice lab, case-based)  Mode of delivery:  Face to face  Environment:  Classroom settings, Philadelphia College of Pharmacy  Schedule:  2-hours of practice lab every other week  2-hours of case studies every other week. | Using EHRs to process inpatient medication orders.  Proficiency in using EHRs for documentation | Individua assessment:  completion of a 3-patient case based including collection, assessment, and plan development through submission handwritten progress note. | 1 of the 3 were graded as pass or fail grade | Students showed statistically significant improvement in their perceptions of preparedness to use EHR in clinical practice.  There was no significant difference in student performance between the group that received EHR training and the group that did not, for both acute and ambulatory patient care APPEs. |
| Skelley 2018 (35), USA | Incorporation of EHRs into an elective course focused on collecting and assessing patient information from the EHR, developing a plan to optimise pharmacotherapy and achieve goals of therapy, implement that plan through documentation within the medical record, and identifying need for monitoring and follow-up. | Expose students to using an EMR in an outpatient setting for patient care activities | Material: the cloud-based electronic health record platform, Practice Fusion  Educational activity:  Blended (Lectures, Cased-based activity)  Mode of delivery:  Face to face  Instructors:  Faculty member (x1) per session  Environment:  Classroom settings, Samford University.  Schedule:  three consecutive class sessions  1^st^: introduction  2^nd:^ 2 Cases total of 1.15 hours  3^rd:^ 2 Cases total of 1.15 hours | Exposures student to digital technology e.g., EHRs.  Using EHRs to find, manage, organise, store, and share patient data. | Group Activity:  Students work in pair, using EHRs to develop and document 4 SOAP (subjective, objective, assessment, plan) notes. | Assessment based on participations in first 2 cases and assessment using rubric in 2 cases. | Pre- and post-survey were completed by 18 students.  Statistically significant changes were observed on three questions:  Pharmacist's skills and knowledge (p=0.046).  Benefits of an EMR in didactic coursework (p=0.046).  Confidence in documenting clinical activities (p<0.001). |
| Darley 2019 (32), USA | A laboratory class focusing on automation equipment was held. Vendor prepared and delivered the lecture, followed by a product demonstration for a group of students. The sessions are led and facilitated by faculty also. | Expose students in-person to current, commercially available digital health technology | Material: automation equipment e.g. automated dispensing cabinets  Educational strategies: skills laboratory class  Modes of delivery: Blended  Instructors: faculty staff (x1) Vendors (x1) per class  Environment:  Classroom settings, the University of Georgia (UGA) College of Pharmacy  Schedule:  30-minute: Presentation and discussion  20-minute: Automated dispensing cabinet demonstration  15-minute: debriefing session with faculty | Exposure to commercially available digital health technologies (automated dispensing cabinets)  Technical proficiency in using automated dispensing cabinets | No assessment details were reported. | | 265 students in the two-year 2017/18 program completed pre- and post-class surveys.  97% of students in both 2017 and 2018 showed an improvement in understanding of automation/informatics products.  89% of students in 2017 and 86% in 2018 showed an improvement in their confidence in using automation/informatics products in the hospital environment. |
| Gibson 2019 (36), USA | Incorporation of a Simulated EHR into Pharmacotherapy course using Microsoft PowerPoint to use in a case-based application course for second-year pharmacy students for two consecutive years. | Increase student proficiency and comfort with the use of EHRs. | Material: Microsoft PowerPoint slides to resemble individual sections of an EMR,  Environment: The classroom setting, University of North Texas System College of Pharmacy  Instructors:  Course faculty x2  Educational strategies: lectures and active learning application exercises  Mode of delivery:  Face to face  Schedule:  10-minute: introduction  90-minute: groups discussion to write a SOAP note using the SEMR | Technical proficiency in using digital technology e.g., EHRs | Group Activity:  Six to seven students work together to write a SOAP note using the simulated EHRs to retrieve subjective and objective information for a simulated case and submit the note within 3 days. | Participation points were given to students who completed both the pre- and post-assessment. | 81 students completed a pre-survey and 162 completed a post-survey.  The results showed statistically significant improvements in perceived confidence (P=0.006) and efficiency navigating EMRs (P=0.004) after the activity. |
| Carr-Lopez 2020 (39), | A module on medication safety developed and piloted, where students Under the direct supervision of VA clinical pharmacists, students accessed veterans' electronic health records (EHRs) and reviewed lab monitoring while working remotely at the school. | Expose students to essential component of clinical care e.g., medication safety.  Provide students a remote access to a health system’s live EHR and real patient data. | Material: The VA Northern California Health Care System (NCHCS), personally owned laptops.  Educational strategies: experiential education  Mode of delivery: Face to face  Instructors:  VA pharmacists or university faculty hired by the VA to perform student precepting for the population health program (x2) per session  Schedule:  2 hrs./ session for six sessions over 3 weeks. | Exposures student to digital technology e.g., EHRs.  Using EHRs to review medication safety. | Individual activity:  Every student was assigned up to five patients to review their patient’s health records using VA system. drafted a progress note. | Preceptor review and approved the students suggested plan. | 58 students completed both pre- and post-survey.  There was a significant improvement in the students' abilities to monitor laboratory tests from 12 to 53 students.  There was also a significant improvement in the students' abilities to use a non-simulated EHR from 10 to 54 students. |
| Ives 2020 (40), | Introducing EHRs to health system-based skills laboratory course to teach Inpatient Medication Order Verification to Pharmacy Students, | Allows students to process inpatient medication orders using technology that simulates real-world processes. | Material: the platform EHR Go! online pre-recorded material  Educational strategies: experiential education  Mode of delivery: Face to face  Instructors:  faculty member (x1) per class  Environment:  Classroom settings, the University of Maryland, School of Pharmacy,  Schedule:  Two practice laboratory sessions | Using EHRs to process inpatient medication orders.  Proficiency in using EHRs for medication verification. | Individual Assignments and  Mid-term practical exam:  students were asked to check one assigned patient with three medication orders and up to three medication errors to decide to verify or flag each order. | Assessment of midterm exam, Students received full credit for either correctly identifying that an individual order should be verified or correctly flagging an order and providing the correct rationale for doing so. | Conducted activity analysis and compared to the previous year.  Significantly increased practical exam mean score (p<0.05) from 69 (n=120) to 83 (n=158) for students who completed activity with paper-based medication form the year before.  Administered pre-and post- surveys to 108 students, resulting in doubled and even tripled confidence levels in EHR use after implementation. |
| O'Connor 2020 (41), | "Exploration of Telemedicine," an online elective course offered to students, gives them the knowledge they need to utilize telehealth in their future practices and opportunity to observe telehealth clinical activities in real time. | Provide a tele-medicine education and hands on clinical experiences. | Educational strategies:  Lectures, discussion and observe telehealth clinical activities in real time  Mode of delivery:  Online  Instructors:  infectious disease physicians, pharmacists, nurses, and caseworker per session.  Environment:  UIC College of Pharmacy | Using digital technologies to communicate with patients.  Technical proficiencies in digital technology utilization e.g., telehealth, EHR | No assessment details were reported. | | Better clinical outcomes observed, including virologic suppression, adherence, and patient quality of life, when compared to traditional in-person management. |
| Cook 2021a (45), | an experience from two pharmacy schools, The University of Nebraska Medical Center (UNMC) and The University of California San Diego (UCSD), where EHRs were integrated into various. Students use the EHR to hone their EHR skills. | evaluate pharmacy students' perceived readiness to utilize the EHR  identify opportunities for curricular design or redesign to address learner needs. | Material: EHRs, EPIC Education (used for training) and EPIC Production (live environment)  Educational strategies:  Case-based, Experiential training and student-run free clinic  Mode of delivery: Face to face  Instructors:  Faculty member, (x1)  Technical staff (x1) per experiential training.  Schedule:  UNMC:  1^st^, 2^nd,^ and 3^rd^ students: integrated EHR into pharmacotherapy skill lab.  2^nd^ and 3^rd^ students: 6 hrs. EHR training  4^th^ year: 4 weeks APPEs  UCSD:  2^nd^ year: hospital pharmacy IPPEs during summer, shadow pharmacist, student-run free clinic  3^rd^ year: 2hrs classroom training  Environment:  classroom and clinical settings. | Using EHRs to find, manage, organise, store, and share patient data.  Proficiency in using EHRs for documentation. | No assessment details were reported. | | Survey completed by 70 UNMC and 69 UCSD students.  Students reported greater comfort in finding information, reviewing laboratory data, progress notes, and medication lists.  Students reported less comfort in performing medication reconciliation and identifying clinical errors. |
| Neumann-Podczaska 2021 (47), Poland, Project report | A remote experimental education project was created and implemented to offer medical and pharmacy students real-world learning experiences while caring for vulnerable older patients (over 60) with very little access to healthcare. | improve student satisfaction with virtual learning.  measure patient acceptance of an interprofessional student telehealth education project | Material: means of communication (telephone, WhatsApp®, or Messenger®), Internet-Telephone Consultation Services (ITCS)  Educational strategies:  mentor-guided discussion, live experience  Instructors: physician (x1)  Pharmacist (x1) per sessions  Environment: Pozna´n University of Medical Sciences (PUMS)  Schedule:  -first remote meeting at a convenient time (six days a week during working hours (8 a.m.–4 p.m.).  -mentor-guided discussion to discuss recommendation  - teleconsultation session. | Using digital technologies to communicate with patients and other healthcare professionals.  Technical proficiencies in digital technology utilization e.g., telehealth, EHR | Students are asked to provide interprofessional medication management, which is conducted by pharmacy and medical students working together under the guidance of their academic mentors. | No assessment for the activity done. | In-depth interviews with students revealed high satisfaction with the IPE experience.  Students reported improvement in self-confidence, patient care skills, literature review ability, communication, telehealth skills, and interprofessional collaboration skills. |
| Perkins 2021 (48),USA | EHR integration was implemented across the pharmacy school curriculum in the first, second, and third professional years.  EHRs were initially piloted in an elective course on Introduction to Internal Medicine before being introduced in several courses | Exposing students to health information technologies used in clinical practice, like electronic health records (EHRs), | Material:  EHR Go  Instructors:  faculty members (x3) per sessions.  Educational strategies:  Case-based  Mode of delivery:  Face to face | Using EHRs to find, manage, organise, store, and share patient data. | Pilot course:  student groups were required to utilize one of the simulated EHR patients to present a patient case in a grand rounds format.  No assessment details were reported afterward. | | Report on EHR utilization showed extensive use, based on overall number of student experiences, range of experiences, and number of faculty using EHR in courses. |
| Wasynczuk and sheehan 2021 (50), | A teaching EHR was incorporated within the required Drug Information (DI) and literature evaluation course. | Introduce students to a teaching EMR to emphasize the importance of patient-centred care.  assess student perceptions of EMR use in the didactic setting. | Material: a web based EHRs platform  Educational strategies: lecture and discussion activities  Modes of delivery: Blended (using online technology)  Environment:  Classroom settings  Schedule:  2 hours/week class, over 12 weeks | Using EHRs to find, manage, organise, store, and share patient data.  Proficiency in using EHRs for documentation. | Patient-specific DI questions consists of two parts: (1) gathering pertinent patient-specific background information; and (2) providing a patient-specific written response within one week. | Students are graded using a standard rubric for:  competency in professionalism, obtaining all relevant patient-specific information, determining that the question was patient specific, obtaining information about the requestor, and for understanding of the context of the question | A small increase in assignment score average from the previous year may indicate a link between perceived benefit and learning outcomes. |

**Supplemental Table 6. constructive alignment components that relate to the learning strategies used in the reviewed studies.**

| Study | Learning objectives | Teaching approach | Mode of assessment | HEE Digital Capabilities |
| --- | --- | --- | --- | --- |
| Mihalas, 1997 (70) | To acquire basic computer skills for pharmacy informatics and pharmacy applications. (70) (skills) | Hands-on experience with software applications used in pharmacy. (70) | The study does not provide specific details on the mode of assessment. (70) | Technical Proficiency  Able to navigate and troubleshoot digital health tools and software with confidence and efficiency. |
| Hincapie, 2016(19) | Demonstrate understanding of basic concepts of health information technology (HIT) and pharmacy informatics.(19) (knowledge) | Team-based learning (TBL) approach, where students worked in teams to solve problems and answer questions related to HIT and pharmacy informatics. (19) | Individual readiness assurance test (iRAT), team readiness assurance test (trat) and application exercises to evaluate and/or analyse several case scenarios around HIT and informatics that pharmacists commonly encounter in pharmacy. (19) |  |
| Ab Rahman, 2002(71) | To provide students with the knowledge necessary to handle drug information.(71) (knowledge) | Lectures (71) | Coursework(40%) essay final examinations (60%) (71) |  |
|  | To equip students with the necessary skills to handle drug information effectively and introduce them to the role of computer-assisted information management in the health care system.(71) ( skills) | A practical session. (71) | Group project, evaluation of pharmacy-related Internet sites and the establishment of group Web sites. (71) | Technical Proficiency  Able to navigate and troubleshoot digital health tools and software with confidence and efficiency. |
| Fuji, 2015 (68) | Ability to Identify health technologies commonly used in the provision of health care, and how these technologies impact the practice of pharmacy.(68) (knowledge) | Online lectures cover these topics.  (68) | Quizzes and Exams assessed students' understanding of course content through multiple-choice, true-false, and short-answer questions administered at regular intervals(68) |  |
|  | Describe the national focus and developments of health information technology and their impact on the pharmacy profession. (68) (knowledge) | Online lectures cover these topics. (68) |  |  |
|  | Identify current problems and future applications for the integration of informatics into pharmacy practice.(68) (knowledge) | Online lectures cover these topics. (68) |  |  |
|  | Demonstrate the ability to discuss health technologies with other health care professionals for the common benefit of safe, quality patient care. (68) (skills) | Discussion forums for students to engage in online discussions and collaborative learning. (68) | Participation in online discussions (68) | Communication, Collaboration and Participation  Able to effectively communicate and collaborate with other healthcare professionals using digital platforms and tools to improve patient outcomes. |
|  | Identify and apply course content to situations in which informatics is commonly used in pharmacy practice (68) (skills) | Group projects and assignments to apply knowledge and skills learned in the course. (68) | Assignments two 5-page literature review, to assess students' ability to apply concepts and skills learned in the course. (68) |  |
| Leibfried, 2016(20) | Develop skills in utilizing Electronic Health Record (EHR) systems such as navigating and documentation using a simulated EMR system.(20) (skills) | Experiential learning through a simulated EMR system in a practice lab setting. (20) | Longitudinal case follow-up and SOAP note documentation, students receive direct observation and feedback from faculty facilitators on ehrs skills. (20) | Information, Data and Content  Able to gather and evaluate relevant health information and data using digital health technology.  Technical Proficiency  Able to navigate and troubleshoot digital health tools and software with confidence and efficiency. |
| Gibson, 2019 | Introduce students to a simulated Electronic Medical Record (EMR) system to practice advanced skills in patient-centred care.(36) (skills) | Using a Microsoft PowerPoint slides to resemble EHRs. (36) | The study lacks clear information regarding the specific method used for assessment.(36) | Technical Proficiency  Able to navigate and troubleshoot digital health tools and software with confidence and efficiency. |
| Skelley,2018 (35) | Demonstrate EHRs proficiency to gather patient information, make informed decisions, and implement care plans aligned with the Pharmacist Patient Care Process (PPCP).(35) (skills) | Hands-on approach where students work to review cases in the EMR and develop SOAP notes. (35) | Three SOAP notes: First ungraded, second had formative feedback with rubric, and final received summative grade with same rubric.(35) | Information, Data and Content  Able to gather and evaluate relevant health information and data using digital health technology.  Technical Proficiency  Able to navigate and troubleshoot digital health tools and software with confidence and efficiency. |
| Brown,2005(53) | To facilitate the documentation and evaluation of simulated patient care activities in pharmacy education using internet based EHRs(53) (skills) | Using an internet-based medical chart for documenting and evaluating simulated patient care activities. (53) | Five "Finish the SOAP Note" activities using an internet-based medical chart allows faculty to evaluate students' documentation and decision-making skills in a simulated patient care setting. (53) | Information, Data and Content  Able to gather and evaluate relevant health information and data using digital health technology.  Technical Proficiency  Able to navigate and troubleshoot digital health tools and software with confidence and efficiency. |
| Brown, 2007(54) | To enhance students' ability to apply pharmacotherapy knowledge to clinical practice by integrating an internet-based medical chart into the lecture series. (54)(skills) | Hands-on approach where students work to review cases in the EMR and develop SOAP notes. (54) | Four "Finish the SOAP Note" activities using an internet-based medical chart allows faculty to evaluate students' documentation and decision-making skills in a simulated patient care setting. (54) | Information, Data and Content  Able to gather and evaluate relevant health information and data using digital health technology.  Technical Proficiency  Able to navigate and troubleshoot digital health tools and software with confidence and efficiency. |
| Smith, 2018(33) | To Familiarise students with EHRs chart navigation and data collection concepts.(33) (knowledge and skills) | Orientation lecture and practical using an EHR through an ungraded learning activity. (33) | NA | Information, Data and Content  Able to gather and evaluate relevant health information and data using digital health technology. |
|  | Provide practice for clinical decision-making and patient care documentation using EHR. (33) (skills) | Completing patient cases during the APPE Preparatory Enrichment course using EHR.(33) | Three written progress notes, two were ungraded, and the third receive pass/fail.(33) | Technical Proficiency  Able to navigate and troubleshoot digital health tools and software with confidence and efficiency. |
|  | Develop competency in medication use through interpretation and application of patient information. (33) (skills) | Complete an acute patient care and ambulatory care APPE. (33) | Comparison of student performance between two groups, one exposed to the simulated EHR and one not exposed, in a didactic setting.(33) | Technical Proficiency  Able to navigate and troubleshoot digital health tools and software with confidence and efficiency. |
| Coons,2018(31) | To integrate patient-specific data using the simulator to develop a pharmacotherapy plan.(31) (skills) | Blended approach with simulated-based learning. Intervention group had virtual EHR access, while control group received baseline info via slides.(31) | Assessment of timing and quality of clinical decision-making for the simulated case using rubric. (31) | Information, Data and Content  Able to gather and evaluate relevant health information and data using digital health technology.  Technical Proficiency  Able to navigate and troubleshoot digital health tools and software with confidence and efficiency. |
| Perkins,2021 | To provide students with hands-on experience in using an EHR system.(48) (skills) | Using EHR platform in various courses with both theoretical and practical learning aspects. (48) | The study lacks clear information regarding the specific method used for assessment. (48) | Information, Data and Content  Able to gather and evaluate relevant health information and data using digital health technology.  Technical Proficiency  Able to navigate and troubleshoot digital health tools and software with confidence and efficiency. |
| Toh,2014 (72) | To provide students with a tool to practice patient record keeping and documentation.  (72) skills | Mobile app simulation-based learning for inputting patient information, reviewing medication history, and documenting patient encounters. (72) | The study lacks clear information regarding the specific method used for assessment. (72) | Information, Data and Content  Able to gather and evaluate relevant health information and data using digital health technology.  Technical Proficiency  Able to navigate and troubleshoot digital health tools and software with confidence and efficiency. |
| Wasynczuk,2021(50) | To effectively use an electronic medical record (EMR) system to answer drug information questions.(50) (skills) | Case-based approach used to teach pharmacy students how to use EMR for drug information inquiry by presenting patient cases with specific circumstances. (50) | Assignment: Gathering patient-specific information and writing a response to a drug-related question.  Part 1: Gathering patient-specific information through a phone call with an instructor and research in the EMR.  Part 2: Writing a response to a drug-related question, graded based on competency criteria such as using relevant literature, identifying patient-specific issues, and providing accurate recommendations.(50) | Information, Data and Content  Able to gather and evaluate relevant health information and data using digital health technology.  Technical Proficiency  Able to navigate and troubleshoot digital health tools and software with confidence and efficiency. |
| Kirwin,2013(62) | To enhance students’ understanding of the role and functioning of EHRs systems in healthcare settings.(62) (Knowledge) | Facilitated discussion about the pharmacist’s role in pharmaceutical care tasks. (62) | Short quiz on concepts related to readings and laboratory activities. (62) |  |
|  | Proficiency in using an electronic medical record (EMR) system to enhance their pharmaceutical care skills. (62) (skills) | Detailed orientation to use of the MEDITECH EMR system. (62)  Simulated EHRs system to follow patients and perform different tasks. (62) | Students were assigned two patients and given the following tasks:  Completion of medication reconciliation worksheets  Compilation of relevant patient information on a monitoring form  Identification and resolution of drug therapy problems using a paper form  Completion of "final check" exercises to verify prepared sterile products  Presentation of patient cases to a laboratory instructor using a standardised format and grading key  Completion of a discharge worksheet to recommend appropriate discharge medication lists and instructions. (62) | Technical Proficiency  Able to navigate and troubleshoot digital health tools and software with confidence and efficiency |
| Cook,2021 (44) | To demonstrate proficiency in using an electronic health record (EHR) system. (44) (skills) | Training approaches at the classroom:  Use of EHR Training Environment (TRN)  Integrated EHRs to pharmacotherapy skills lab and course  Classroom training for students who volunteer at the student-run free clinic  Training approaches during APPEs:  Formal EHR training provided by staff from the academic medical centre  Interaction with live EHRs during Advanced Pharmacy Practice Experiences (APPEs)  Orientation and FT on the EHR during hospital pharmacy IPPEs  Shadowing pharmacists and other healthcare professionals to learn about EHRs role as a hospital-based pharmacist. (44) | The study lacks clear information regarding the specific method used for assessment.(44) | Information, Data and Content  Able to gather and evaluate relevant health information and data using digital health technology.  Technical Proficiency  Able to navigate and troubleshoot digital health tools and software with confidence and efficiency. |
| Carr-lopez 2020.(39) | Ability to review patients health records to improve their medication safety using remote access EHRs(39) (skills) | Didactic instruction on medication safety  A demonstration of remote access to Veterans' health records(39)  Hands-on practice accessing and reviewing the records. (39) | A competency-based assessment to evaluate pharmacy students' medication safety skills. The assessment includes tasks such as logging into the VA system, navigating the EHR, finding medication profiles and progress notes, and reviewing lab results and continuity of care documents. Students must also document their findings accurately in the VA's EHR. (39) | Technical Proficiency  Able to navigate and troubleshoot digital health tools and software with confidence and efficiency. |
| Frenzel,2010 (59) | Able to use EHRs in disease state management activities to facilitate patient-centred care (59) (skills) | Laboratory activities focused on disease state management, longitudinally followed one simulated case using EHRs. (59) | Written a SOAP note and assessed for accuracy of recommending treatment.(59) | Information, Data and Content  Able to gather and evaluate relevant health information and data using digital health technology.  Technical Proficiency  Able to navigate and troubleshoot digital health tools and software with confidence and efficiency. |
| Sei  Fert,2004(52) | Understand the role of a tele pharmacist in providing healthcare services to rural communities(52) (knowledge and skills) | Combination of didactic coursework, and experiential learning included shadowing experienced tele pharmacists and interacting with patients over the phone. (52) | Quality of documentation of a daily log sheet of their activity.  Live evaluations of student performance in Telecommunication stations by the faculty mentor. (52) | Communication, Collaboration and Participation  Able to effectively communicate and collaborate with other healthcare professionals using digital platforms and tools to improve patient outcomes.  Technical Proficiency  Able to navigate and troubleshoot digital health tools and software with confidence and efficiency |
| Estes,2016(18) | Familiarise students with telehealth monitoring tools and their usage, enabling them to utilise these tools effectively in patient care.(18) (skills) | Simulate an IP telehealth visit using videoconferencing, monitoring tools, and academic EHR. (18) | The study lacks clear information regarding the specific method used for assessment.(18) | Information, Data and Content  Able to gather and evaluate relevant health information and data using digital health technology. |
|  | Provide hands-on experience to students in conducting a telehealth encounter in interprofessional clinical environments.(18) (skills) |  |  | Technical Proficiency  Able to navigate and troubleshoot digital health tools and software with confidence and efficiency |
|  | Improve inter-professional collaboration and communication skills among students.(18) (skills) |  |  | Communication, Collaboration and Participation  Able to effectively communicate and collaborate with other healthcare professionals using digital platforms and tools to improve patient outcomes. |
| Neumann-podczaska, 2021(47) | To provide inter-professional telehealth experience for students.(47) (skills) | An interprofessional telehealth education project. (47) | The study lacks clear information regarding the specific method used for assessment.(47) | Technical proficiency  Able to navigate and troubleshoot digital health tools and software with confidence and efficiency.  Communication, collaboration and participation  Able to effectively communicate and collaborate with other healthcare professionals using digital platforms and tools to improve patient outcomes. |
| Fox,2017(25) | Understand the principles and applications of mobile health (mHealth) technologies. (25) (knowledge and skills) | Introduce a course on mHealth using IP approach included various activities. (25) | Various assignments were used Include:  Course proposals on mHealth, purchase plans for mHealth lab equipment, development of an mHealth application. (25) |  |
|  | Ability to communicate mobile technology needs and solutions with software engineers students.(25) (skills) | Active and collaborative learning were used in developing a course proposal and presentation around mHealth (25) | Course proposals and Presented 30-minute "pitch"  Presentations on proposed courses.(25) | Communication, Collaboration and Participation  Able to effectively communicate and collaborate with other using digital platforms and tools to improve patient outcomes. |
|  | Develop practical skills for designing and implementing mHealth interventions. (25) (skills) | Project-based learning was used for purchase plan for mHealth technology  Problem-based learning was used in app prototype. (25) | Developed purchase plans for mHealth lab equipment,  Development of an mHealth application. (25) | Creation, Innovation and Research  Able to create and innovate digital health solutions to improve patient care and outcomes and conduct research using digital health technology. |
| Miranda,2016(21) | Introduce mHealth technology and the process of evaluation and implementation to pharmacy students.(21) (knowledge and skills) | Lectures cover topics such as mHealth basics, evaluating mHealth apps and devices, and implementing mHealth programmes in pharmacy practice. (21)  Hands-on activities include downloading and evaluating mHealth apps, and developing a plan for implementing an mHealth program in a hypothetical pharmacy setting. (21) | Worksheet for students to evaluate specific mHealth devices. (21) | Information, Data and Content  Able to evaluate relevant health information and data using digital health technology. |
| Rodis,2016(16) | Equip pharmacy students with the skills to find, evaluate, and use medical apps. (16) (skills) | Pre-recorded lecture covers the role of mobile medical apps in clinical practice, issues, and opportunities with utilization of medical apps, and how to review and assess medical apps  Workshop, and project-based experiences focused on evaluation of medical apps (16) | Evaluate medical apps using the evaluation tool and presented their findings to the class(16) | Information, Data and Content  Able to evaluate relevant health information and data using digital health technology. |
| Zagar,2007(55) | Describe standard and low-income provisions of Medicare Part D.(55) (Knowledge)  List formulary requirements for Medicare Part D plans.(55) (Knowledge) | Lecture-based instruction on the Medicare Part D program and its options. (55) | Students work in groups to create a list of "The Top Ten Things Every Medicare Beneficiary Should Know About Medicare Part D" and present it to the class. (55) | Information, Data and Content  Able to gather and evaluate relevant health information and data using digital health technology. |
|  | Ability to Assist patients in evaluating and enrolling in Medicare Part D plans.(55) (Skills) | Facilitated online navigation through the Medicare Prescription Drug Plan Finder available on the Medicare web site. (55)  Mock counselling session to apply the information they learned.(55) | Written paper comparing the 3 plans and justifying their choice for the individual assignment. (55)  Mock counselling | Technical Proficiency  Able to navigate and troubleshoot digital health tools and software with confidence and efficiency. |
| Cutler,2008(56) | Improving students' understanding of Medicare Part D.(56)(knowledge) | Lectures covering basic concepts on Medicare part D (56) | NA |  |
|  | Enhance student' skill and confidence in using the online Medicare Prescription Drug Plan Finding tool.(56) (skills) | A Problem-based learning (PBL) session include case studies that discussed using the actual online tool. (56) | A case-based exercise before and after the course to Identify correctly the 3 least expensive plans and corresponding costs. Assessed based on the accuracy of the plan and/or the cost (56) | Technical Proficiency  Able to navigate and troubleshoot digital health tools and software with confidence and efficiency. |
| Darley ,2019(32) | To enhance student knowledge of informatics through in-person exposure to HIT equipment commonly used in hospital settings to provide patient care.(32) (Knowledge) | Introducing an informatics skills laboratory involved Lecture covering topics such as, types of automation equipment and the role of technology in the medication use process, pharmacy operations, quality and safety of care, and cost reduction, and a demonstration. (32) | The study lacks clear information regarding the specific method used for assessment. (32) |  |
| Pantazi,2011(69) | Ability to complete a clinical workflow using a hospital information system (HIS).(69) skills | Incorporation of a hospital information system (HIS) involve simulations and role playing for clinical workflow simulation. (69) | Practical exams on student ability to complete clinical workflows focus on clinical and administrative tasks related to pharmacy management. (69) | Technical Proficiency  Able to navigate and troubleshoot digital health tools and software with confidence and efficiency. |
| Ives,2020(40) | Ability to verify inpatient medication orders using electronic health record (EHR) technology.(40) skills | Implementation of EHR simulation in health system-based skills laboratory course (40) | One assigned patient with three medication orders and up to three medication errors was the subject of the practical examination. (40) | Information, Data and Content  Able to gather and evaluate relevant health information and data using digital health technology.  Technical Proficiency  Able to navigate and troubleshoot digital health tools and software with confidence and efficiency. |
